# Sigma Factor SigB Is Crucial to Mediate *Staphylococcus aureus* Adaptation during Chronic Infections

**DOI:** 10.1371/journal.ppat.1004870

**Published:** 2015-04-29

**Authors:** Lorena Tuchscherr, Markus Bischoff, Santiago M. Lattar, Mariangeles Noto Llana, Henrike Pförtner, Silke Niemann, Jennifer Geraci, Hélène Van de Vyver, Martin J. Fraunholz, Ambrose L. Cheung, Mathias Herrmann, Uwe Völker, Daniel O. Sordelli, Georg Peters, Bettina Löffler

**Affiliations:** 1 Institute of Medical Microbiology, Jena University Hospital, Jena, Germany; 2 Institute of Medical Microbiology and Hygiene, University of Saarland Medical Center, Homburg, Germany; 3 Instituto de Investigaciones en Microbiología y Parasitología Médica (IMPaM UBA-CONICET) y Facultad de Medicina, University of Buenos Aires, Buenos Aires, Argentina; 4 Institute of Genetics and Functional Genomics, University Medicine Greifswald, Greifswald, Germany; 5 Institute of Medical Microbiology, University Hospital of Münster, Münster, Germany; 6 Department of Microbiology, Biocenter, University of Würzburg, Würzburg, Germany; 7 Dartmouth Medical School, Hanover, New Hampshire, United States of America; The University of Texas-Houston Medical School, UNITED STATES

## Abstract

*Staphylococcus aureus* is a major human pathogen that causes a range of infections from acute invasive to chronic and difficult-to-treat. Infection strategies associated with persisting *S*. *aureus* infections are bacterial host cell invasion and the bacterial ability to dynamically change phenotypes from the aggressive wild-type to small colony variants (SCVs), which are adapted for intracellular long-term persistence. The underlying mechanisms of the bacterial switching and adaptation mechanisms appear to be very dynamic, but are largely unknown. Here, we analyzed the role and the crosstalk of the global *S*. *aureus* regulators *agr*, *sarA* and SigB by generating single, double and triple mutants, and testing them with proteome analysis and in different *in vitro* and *in vivo* infection models. We were able to demonstrate that SigB is the crucial factor for adaptation in chronic infections. During acute infection, the bacteria require the simultaneous action of the *agr* and *sarA* loci to defend against invading immune cells by causing inflammation and cytotoxicity and to escape from phagosomes in their host cells that enable them to settle an infection at high bacterial density. To persist intracellularly the bacteria subsequently need to silence *agr* and *sarA*. Indeed *agr* and *sarA* deletion mutants expressed a much lower number of virulence factors and could persist at high numbers intracellularly. SigB plays a crucial function to promote bacterial intracellular persistence. In fact, Δ*sigB*-mutants did not generate SCVs and were completely cleared by the host cells within a few days. In this study we identified SigB as an essential factor that enables the bacteria to switch from the highly aggressive phenotype that settles an acute infection to a silent SCV-phenotype that allows for long-term intracellular persistence. Consequently, the SigB-operon represents a possible target to develop preventive and therapeutic strategies against chronic and therapy-refractory infections.

## Author Summary

*Staphylococcus aureus* is a frequent pathogen of severe invasive infections that can develop into chronicity and become extremely difficult to eradicate. Chronic infections have been highly associated with altered bacterial phenotypes, i.e., the small colony variants (SCVs) that dynamically appear after bacterial host cell invasion and are highly adapted for intracellular long-term persistence. In this study, we analyzed the underlying mechanisms of the bacterial switching and adaptation process by investigating the functions of the global *S*. *aureus* regulators *agr*, *sarA* and SigB. We demonstrate that a tight crosstalk between these factors supports the bacteria at any stage of the infection and that SigB is the crucial factor for bacterial adaptation during long-term persistence. In the acute phase, the bacteria require active *agr* and *sarA* systems to induce inflammation and cytotoxicity, and to establish an infection at high bacterial numbers. In the chronic stage of infection, SigB downregulates the aggressive bacterial phenotype and mediates the formation of dynamic SCV-phenotypes. Consequently, we describe SigB as a crucial factor for bacterial adaptation and persistence, which represents a possible target for therapeutic interventions against chronic infections.

## Introduction

*S*. *aureus* is a major human pathogen that can infect almost every organ in the body and cause destructive infections [1]. Besides tissue damage the ability to develop persisting and therapy-refractory infections poses a major problem in clinical practice, such as endovascular and bone infections [2,3]. Chronic infections require prolonged antimicrobial treatments and can have a dramatic impact on the patients`quality of life, as they often afford repeated surgical interventions with the risk of amputation or loss of function [2,4].

To induce an infection *S*. *aureus* expresses a multitude of virulence factors, including surface proteins and secreted components, like toxins and peptides [1]. Toxins and other secreted factors are mainly directed against invading immune cells, but can also cause tissue damage that enables the bacteria to enter deep tissue structures [5,6]. Yet, *S*. *aureus* is not only an extracellular pathogen, but can also invade a wide variety of mammalian cells, such as osteoblasts, epithelial- and endothelial cells [7–9]. All cells discussed in the literature as “non-professional phagocytes” possess mechanisms that nevertheless permit endocytotic uptake and degradation of microorganisms [10–12]. To evade the intracellular degradation machineries the bacteria have evolved different strategies, such as the killing of host cells or the escape from the lysosomal compartments and silent persistence within the intracellular location [8,13,14]. Only recently, we demonstrated that *S*. *aureus* can dynamically switch phenotypes from a highly aggressive and cytotoxic wild-type form to a metabolically inactive phenotype (small colony variants, SCVs) that is able to persist for long time periods within host cells without provoking a response from the host immune system [9]. In their intracellular location the bacteria are most likely very well protected from antimicrobial treatments and the host´s defense system. This is a possible reservoir for chronic and recurrent infections. Yet, the environmental changes encountered by invading bacteria during the passage from an extracellular to the intracellular milieu and during long term persistence within the intracellular shelter most likely cause diverse stress conditions. The adaptation mechanisms involved and how bacteria cope with this stress, are largely unknown, but probably involve global changes in gene expression to promote survival.

It is well known that *S*. *aureus* possess a large set of regulatory factors that control the expression of virulence determinants [15–18]. A very important and well-studied system is the accessory gene regulator (*agr)*-locus with the effector molecule RNA III [19,20]. Many *S*. *aureus* derived factors that induce inflammation or cell death are under the control of this system. Important cytotoxic components regulated by the *agr*-system are the pore-forming *α*-hemolysin (*α*-toxin, Hla) and the phenol-soluble modulins (PSMs), which are strong cytotoxic and pro-inflammatory factors in different host cell types [6,21–23]. SarA, which is the major protein encoded by the *sar*-locus, is believed to contribute to the activation of *agr* expression [24,25]. This is supported by findings of many *S*. *aureus* infection models demonstrating that mutations of either loci result in attenuation of virulence [26–28]. Beyond that, it has been shown that SarA influences the regulation of several virulence factors independently of *agr*, e.g. expression of adhesins [25,29,30]. The alternative sigma factor B (SigB; σ^B^) modulates the stress response of several Gram-positive bacteria, including *S*. *aureus* [31–33]. SigB is responsible for the transcription of genes that can confer resistance to heat, oxidative and antibiotic stresses [16,31,34,35]. The sigB system is linked to the complex *S*. *aureus* regulatory network, as it increases *sarA* expression, but decreases RNA III production [36].

During the infection process the bacteria encounter different stressful conditions that they need to overcome in order to settle and maintain an infection. In the acute infection they have to fight against invading immune cells and destroy tissue cells to enter deep tissue structures, whereas during the chronic phase a major challenge is most likely the hostile intracellular location deprived of nutrients and lysosomal degradation. Consequently, the host-pathogen interaction needs to be very dynamic. Yet, to date no studies have followed the adaptation mechanisms and the impact of regulators during the whole infection process. In this work we focused on the interaction of the global regulatory systems *agr*, SarA and SigB and we demonstrate a crucial function for SigB in bacterial adaptation mechanisms and the formation of SCV-phenotypes.

## Results

### Agr and SarA are required for inflammation and cytotoxicity

**Fig 1:**
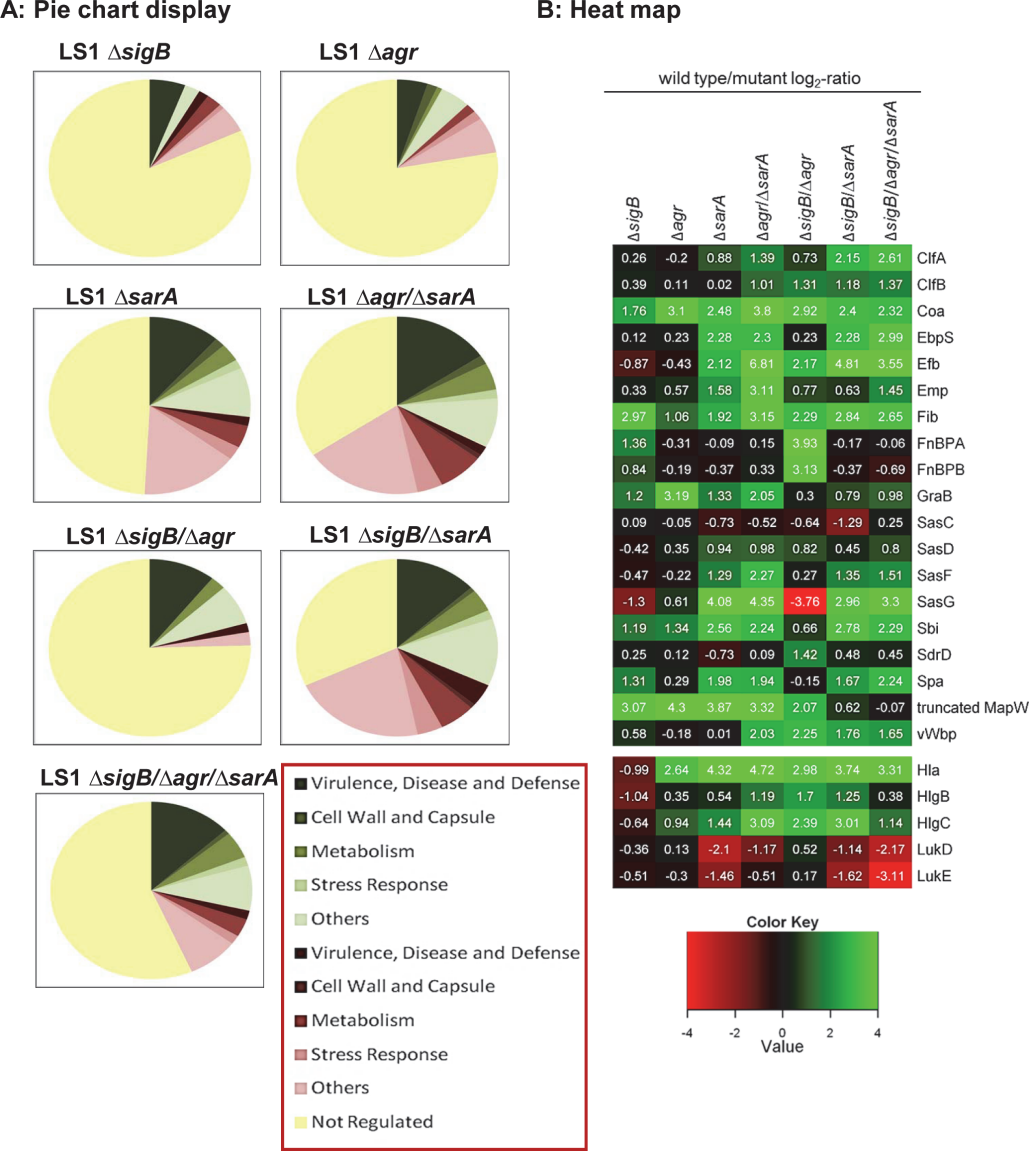
Proteomic analysis of strain LS1 and corresponding mutants. (A) Pie chart display of the proportion of secreted/ cell surface proteins displaying alterations in level in the different mutants compared to the wild type strain. Only proteins with SEED annotation are included and they are categorized according to SEED. Proteins displaying reduced and increased levels in the mutants compared to the wild type are displayed in shades of green and red, respectively. (B) Heat map of proteins which belong to the groups of adhesive and surface proteins or to secreted toxins. Log_2_-values of the ratios of intensity values of the wild type divided by those of the mutants from -4 to +4 are shown. Higher expression in the wild type versus the mutants is displayed in shades of green and higher expression in the mutants compared to the wild type in shades of red. Proteins displaying the same intensities in wild type and mutant are colored black.

For our study we generated single mutants for the functions of SigB (Δ*sigB*), *agr* (Δ*agr*), SarA (Δ*sarA*) and the complemented mutant for sigB (Δ*sigB* compl.), three double mutants for SigB, *agr*, SarA (Δ*agr*/Δ*sarA*, Δ*sigB/*Δ*agr*, Δ*sigB/*Δ*sarA*) and a triple mutant (Δ*sigB/*Δ*agr*/Δ*sarA*) in *S*. *aureus* LS1, a strain derived from mouse osteomyelitis [37]. Several mutants were also generated in the *rsbU* complemented derivative of the laboratory strain 8325–4, SH1000 [33] (Supp. S1 Table). All strains and mutants were characterized by growth curves, which did not reveal any substantial differences between mutants and parent strains (S1 Fig). Yet, differences in hemolysis were present in many mutants, particularly in the *agr-*, *sarA-*, double and triple mutants, which exhibited low hemolysis (S1C Fig). Furthermore, the strain LS1 and the corresponding mutants were analyzed by LC-MS/MS mass spectrometry, which was focused onto the culture supernatants to provide an overview on the levels of virulence factors in each strain (Fig 1A and S3 Table). These data show that particularly the *sarA*-mutant and even more the double and the triple-mutants released a much reduced number of virulence factors associated with disease development compared with the wild-type strain LS1 (reduced levels of virulence factors associated with disease; deep green areas, Fig 1A); in particular different adhesive proteins and toxins were present in reduced levels in culture supernatants (Fig 1B). Yet, for the FnBPs that are important for host cell invasion we detected similar levels compared with the wild-type LS1 in most mutants that can account for the invasive capacity of the strains in host cells (S5 Fig). Most importantly, the *sigB*-mutant showed only modest alterations in protein levels (Fig 1A), but increased levels of *α*-toxin that is known as a strong proinflammatory and cytotoxic factor after host cell invasion (*hla*, Fig 1B). The other listed toxins, e.g. lukD and lukE, are reported as non-hemolytic and only poorly leucotoxic toxins [37].

**Fig 2:**
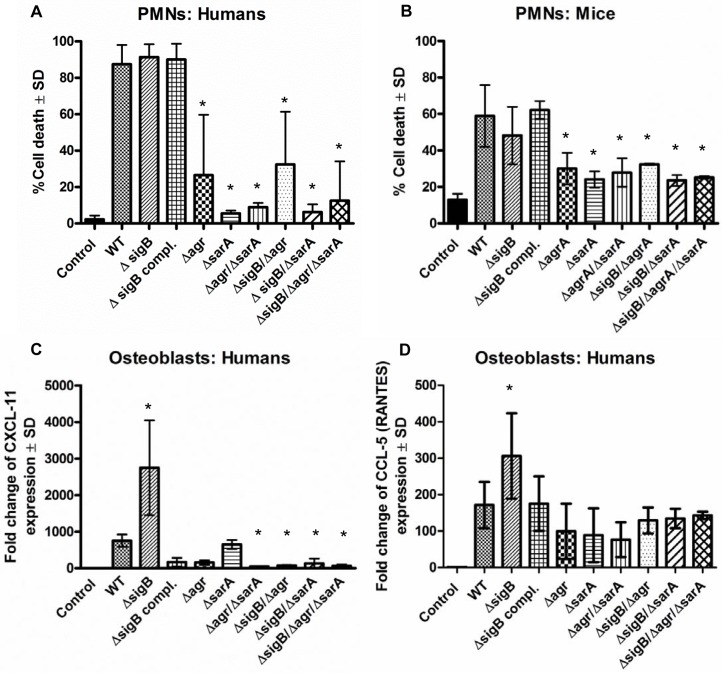
The combined action of *agr* and *sarA* is required for inflammation and cytotoxicity in the acute stage of infection. Cytotoxicity experiments and analysis of inflammatory host response were performed in polymorphonuclear cells (PMNs), human osteoblasts and endothelial cells using wild-type strain LS1 and their derivate mutants. (A, B) PMNs were freshly isolated form human blood (A) and bone marrow of Balb/C mice (B) and 1×10^6^/0.5 ml cells were incubated with 5% v/v of bacterial supernatants for 1 h. Then cells were washed, stained with annexin V and propidium iodide and cell death was measured by flow cytometry. (C, D) Cultured osteoblasts were infected with *S*. *aureus* LS1 or their derivate mutants (MOI 50). After bacterial invasion (3 h) extracellular staphylococci were removed by washing and lysostaphin treatment and infected cells were incubated with culture medium for 48 h. To analyze host cell response the changes in the expression of the chemokine CXCL-11 and CCL-5 were measured by real-time PCR. Results demonstrate the relative increase in gene expression, compared to unstimulated cells (control: expression 1). The fold change is the normalized expression of each gene to housekeeping genes (β-actin and GAPDH). The values of all experiments represent the means ± SD of at least three independent experiments performed in duplicates. * P≤0.05 ANOVA comparing the effects induced by the wild-type strains and the corresponding mutants. Similar results were obtained with strain SH1000 and selected mutants (S2 Fig and S3 Fig).

As secreted virulence factors are particularly directed against professional phagocytes, we tested the effect of bacterial supernatants on neutrophils (PMNs) isolated from humans and mice. In line with the proteomic data, only the strains with high levels of virulence factors (LS1, Δ*sigB*, Δ*sigB* compl.) caused cell death, whereas all other mutants with reduced levels of toxins induced significantly less cytotoxicity (Figs 2A and 2B and S2A–S2C). This effect was concentration dependent and revealed highest levels of cell death in response to supernatants of the sigB-mutant (S2C and S2D Fig). Next we analyzed levels of chemokine expression in cultured tissue cells, such as osteoblasts and endothelial cells, 48 h after infection by real time PCR (Fig 2C and 2D and S4C and S4D Fig) and 24 h after infection by ELISA-measurements (S3C and S3D Fig). In contrast to the wild-type strain, all double-and triple-mutants (including mutations in SigB) exhibited reduced cell activating activity, whereas the *sigB*-single-mutant often caused even more cell activation than the wild-type strain. Furthermore, these effects were independent of the bacterial background and of the infected host cell types, as they were reproduced with selected mutants generated in strain SH1000 (S3D Fig). All effects were equally present in bone and endothelial cells (S4C and S4D Fig) regardless of the observation that endothelial cells took up higher amounts of bacteria than osteoblasts (S4A Fig). Nevertheless, endothelial cells expressed in general lower levels of chemokines than osteoblasts (S4B Fig).

Taken together, these results suggest that the *agr*- and SarA-systems are required to mount an aggressive and cytotoxic phenotype during acute infection, while SigB appears to have a restraining function on virulence. Nevertheless, since double and triple mutants are weak in virulence, the interaction of SigB with the *agr*- and SarA-systems is not clear during acute infection.

### SigB is required for SCV-formation, whereas the *agr*- and SarA-systems need to be silenced for intracellular persistence

**Fig 3:**
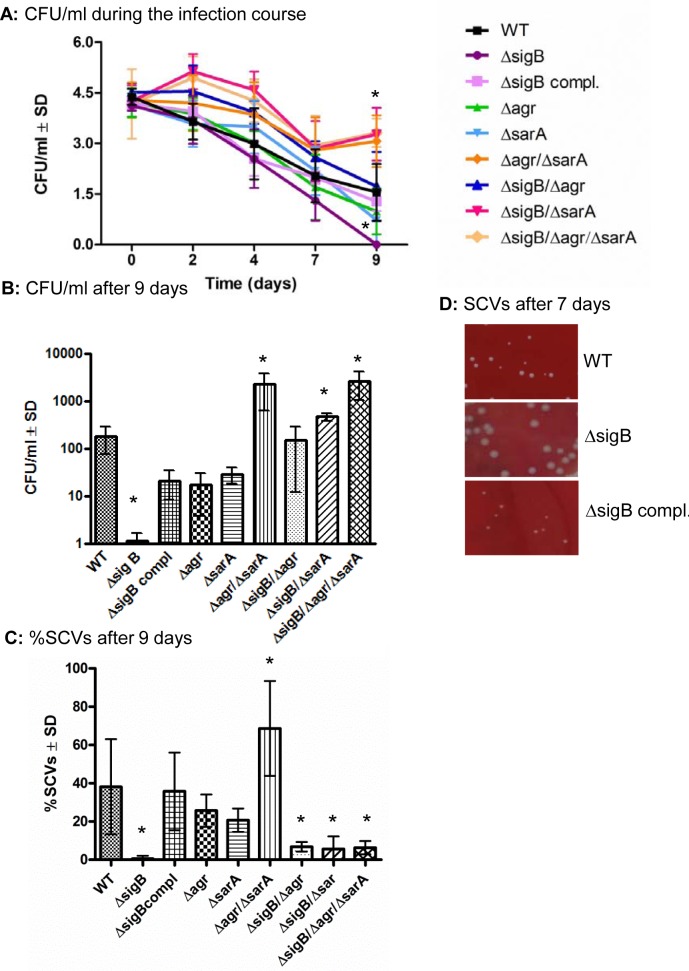
*sigB* promotes persistence and SCV-formation. (A) Cultured osteoblasts were infected with *S*. *aureus* strain LS1 or the corresponding or complemented mutants and infected cells were analysed for up to 9 days. The numbers of viable intracellular persisting bacteria were determined every 2 days by lysing host cells, plating the lysates on agar plates and counting the colonies that have grown on the following day. (B) The results after 9 days are demonstrated separately. The results shown here are from osteoblast infection experiments, but similar results were obtained after infection of endothelial cells. (C) The percentage of small and very small (SCV) phenotypes (<5 and <10-fold smaller than those of the wild-type phenotypes, respectively) recovered (between 200 and 500 colonies examined in each sample) were determined after 7 days p.i. The values of all experiments represent the means ± SD of at least three independent experiments performed in duplicates. * P≤0.05 ANOVA test comparing the effects induced by the wild-type strains and the corresponding mutants. (D) Photographs of recovered colonies were performed after 7 days of infection of endothelial cells with strains LS1, LS1∆*sigB* or LS1∆*sigB* compl. Similar results were obtained when osteoblasts were infected with SH1000 and selected mutants (S7 Fig).

To test the function of the global regulatory systems in the course from acute to chronic infection, we infected osteoblast and endothelial cell cultures with wild-type and mutant strains and analyzed their ability to persist intracellularly for 9 days. All strains were invasive in osteoblasts to a similar extent (S5 Fig) and induced cell death ranging around 50% immediately after infection (S6A and S6B Fig). Yet, in the following 2–3 days the integrity of the infected cell monolayers were fully recovered and the rate of cell death was reduced to control levels (S6C Fig). In general the numbers of intracellular bacteria were decreased during the whole infection course (Figs 3A and S7A), but considerable differences between the strains appeared after several days (9 days, Figs 3B and S7B). The *agr*/*sarA-* and the *sigB/sarA*-double mutants as well as the triple mutant were able to persist within the intracellular location at significantly higher numbers (up to 100-fold) than the corresponding wild-type strain (Fig 3A and 3B). By contrast, the *sigB*-mutants were completely cleared from the host cells within 7–9 days, whereas this effect could be fully reversed by the complementation of *sigB* (Figs 3A and S7A). To test whether this effect is specific for *sigB*-mutants, we further tested mutants for other virulence or regulatory factors such as for *sae* and *hla* which did not reveal any differences in the numbers of intracellular persisting bacteria compared with the wild-type strain (S8 Fig). From our previous work we know that bacterial persistence is associated with dynamic SCV-formation. As recently described [9] we discovered an increased rate of SCV-formation after several days of intracellular bacterial persistence, whereby the recovered SCV were not stable, but the majority reverted back to the wild-type phenotype upon 2 to 5 subcultivating steps on agar plates. Interestingly, in the present study we found that all *sigB*-mutants completely failed to develop SCV phenotypes after 7 days of intracellular persistence (Fig 3C and 3D and S7C and S7D Fig). By analyzing the recovered colonies from *sigB*-mutants, we observed much less phenotypic diversity than in the wild-types and other mutants, as the plates revealed only uniform large white colonies. Again these effects could be reversed by complementation of the *sigB*-mutations with an intact *sigB*-operon, thus proving a clear and specific connection between the bacterial ability to form dynamic SCVs and the SigB-system.

**Fig 4:**
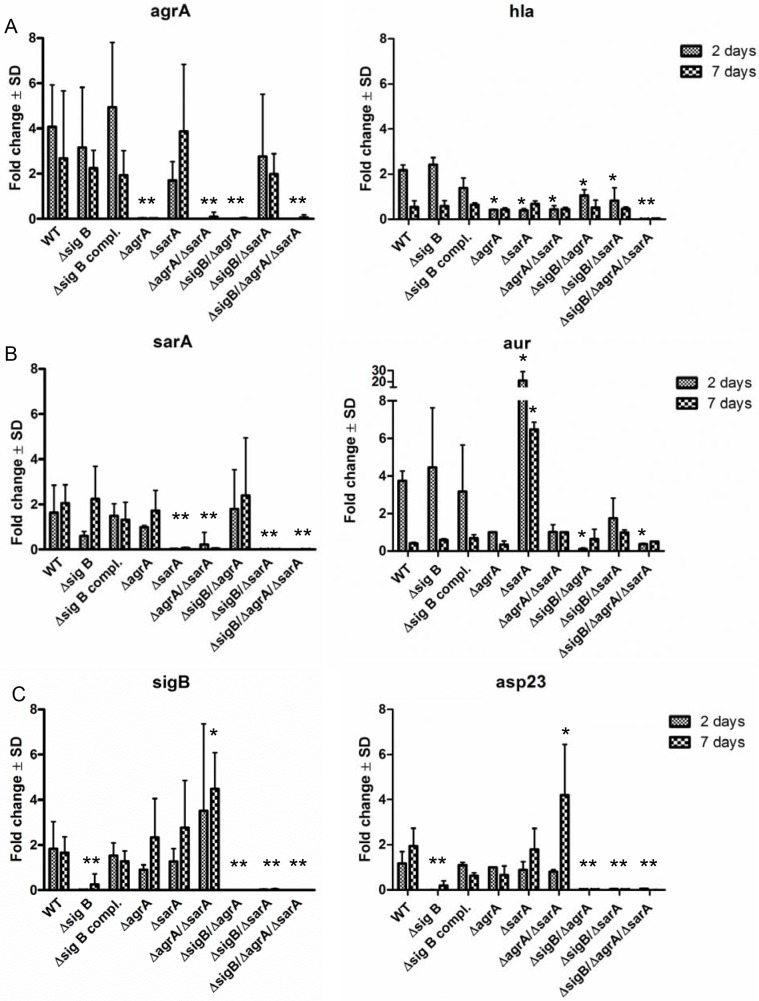
Differential expression of the regulators *agr*, *sarA* and *sigB* during the course of host cell infection in wild-type LS1 and the corresponding mutants. Endothelial cells were infected with *S*. *aureus* strain LS1 or the corresponding mutants as described in Fig 3A and infected cells were analysed for up to 7 days. Host cells infected with wild-type strain LS1 were lysed after 2 (acute phase) and 7 days (chronic phase) and the whole RNA was extracted and was used to determine changes in bacterial gene expression for *agrA/hla* (α-hemolysin) (A), *sarA/aur* (aureolysin) (B) and *sigB/asp23* (C) during the course of infection by real-time PCR. The values of all experiments represent the mean ± SD of 5 independent experiments measured in triplicate. * P≤0.05 ANOVA comparing levels of gene expression in the wild-type strains and the corresponding mutants at each time point. The fold change is the result of normalized expression to the housekeeping genes *gyr*, *aroE* and *gmk*. Uninfected cells were used as controls (Control = 1).

To further explain the differential ability of the mutants to persist, we evaluated the expression of the global regulatory systems during the long course of the infection. To accomplish this, we extracted RNA from infected host cells (HUVEC; as they can host higher numbers of bacteria, S4A Fig) at day 2 and day 7 p.i. and measured the expression of *agr*, *sarA* and *sigB* and the related factors *hla* (regulated by *agrA*), *aur* (repressed by *sarA* [38]) and *asp23* (regulated by *sigB* [39]) in the wild-type strain and corresponding mutants by quantitative real-time PCR (Fig 4). As expected high levels of both *agrA* and *sarA* were only expressed by the strains LS1, Δ*sigB* and Δ*sigB* compl. that resulted in high levels of *hla* expression in the acute phase of the infection. By contrast, the *agr*/*sarA*-double mutant expressed *sigB* and *asp23* at significant higher levels than the wild-type strain during chronic infection (Fig 4) and was able to form higher numbers of SCV phenotypes (Figs 3C and S7C and S7D). Taken together, our results show that a concomitant downregulation of *agrA* and *sarA* promotes long-term intracellular persistence of *S*. *aureus*. SigB promotes chronic infections and is highly associated with the bacterial ability to form SCVs.

### An active *agr* and/or SarA-system is required for lysosomal escape after host cell invasion

**Fig 5:**
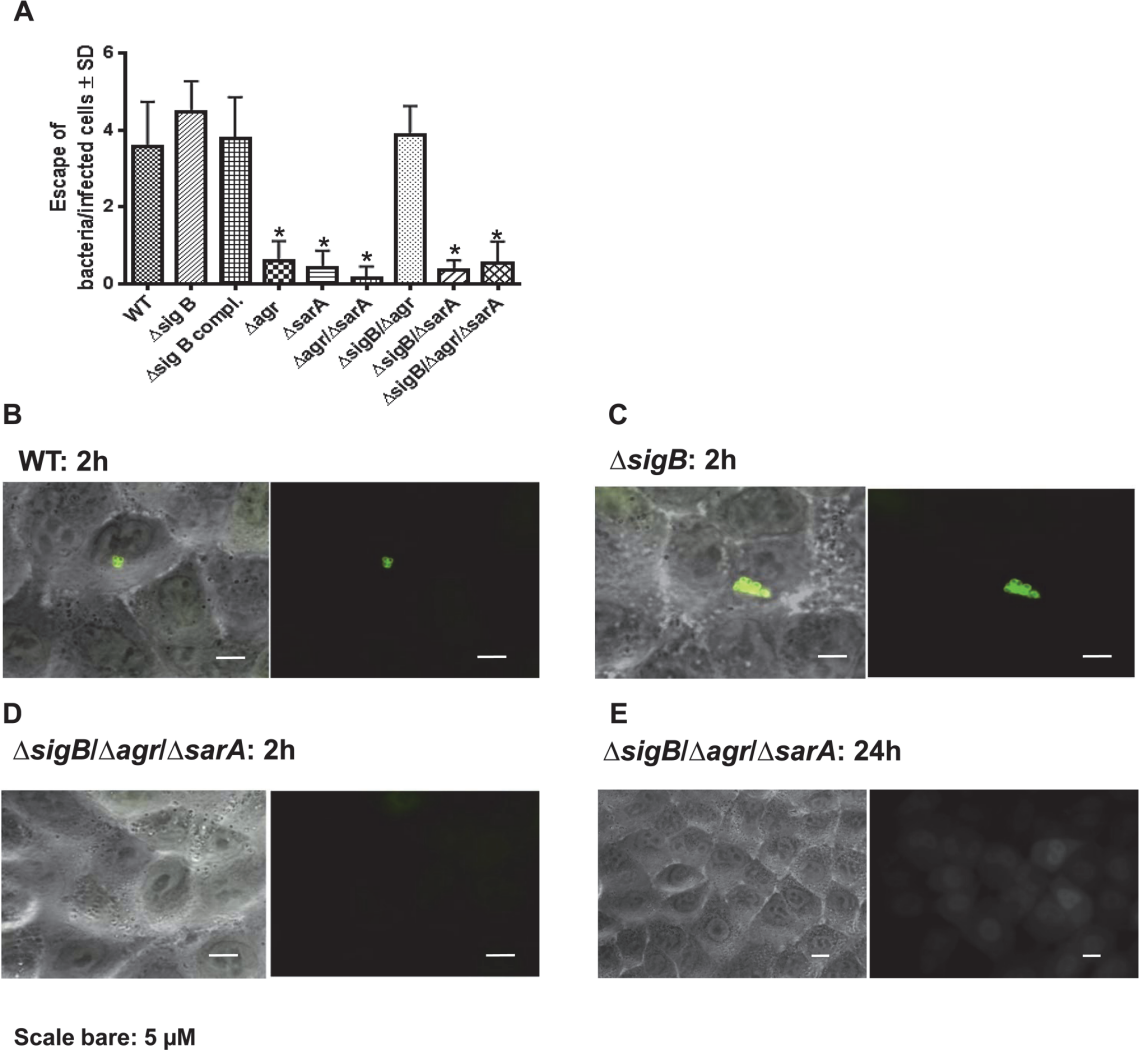
Phagosomal escape of wild-type strain LS1 and corresponding mutants after infection of the host cell construct A549YFP-CWT. Phagosomal escape was measured 2 hours (A, B, C, D) and 24 hours (E) after infection with the strain LS1 or corresponding mutants in genetically engineered A549 cells. This reporter recruitment technique has been recently described [13]. To quantify the phagosomal escape for each strain 10 fields of view were evaluated respectively and the numbers of escaping bacteria were counted. The values represent the mean ± SD of 5 independent experiments measured.* P≤0.05 ANOVA comparing levels of gene expression in the acute and chronic stage.

Only recently, phagosomal escape to the cytoplasm was reported for different *S*. *aureus* strains early after host cell invasion [13,40]. In a further approach we analyzed whether an early phagosomal escape is a prerequisite for persistence. Therefore we used a reporter recruitment technique based on the host cell line A549 genetically engineered to produce a phagosomal escape marker [13]. Within the first 2 h after host cell infection we detected phagosomal escape for the wild-type strain LS1, the *sigB*-, the *sigB* compl.- and the *sigB/agr*-mutants (Fig 5A–5D). These strains showed only weak changes and down-regulation of virulence factor expression by proteomic analysis compared with the wild-type (Fig 1A) and were not able to persist at high bacterial numbers (Fig 3B). As the single *agr* and *sarA* mutants readily lost their ability to translocate to the cytoplasm, apparently both *agr*- and *sarA*-regulated factors are required for the escape mechanism. The activity of SarA alone is sufficient only in case of a non-functional SigB-system, indicating a modulating role of SigB in virulence factor expression. Our results show that an early phagosomal escape is not required for persistence. Further on, mutants that persisted at high bacterial numbers did not escape to the cytoplasm. As phagosomal escape could not be detected at later stages of infection (up to 24 h; as shown in for the triple mutant; Fig 5E), it must be assumed that these mutants are not degraded within phagolysosomes and thus persist in phagosomes at high bacterial numbers.

### The combined dynamic action of the global regulators *agr*, SarA and SigB is required to efficiently establish an infection *in vivo*

**Fig 6:**
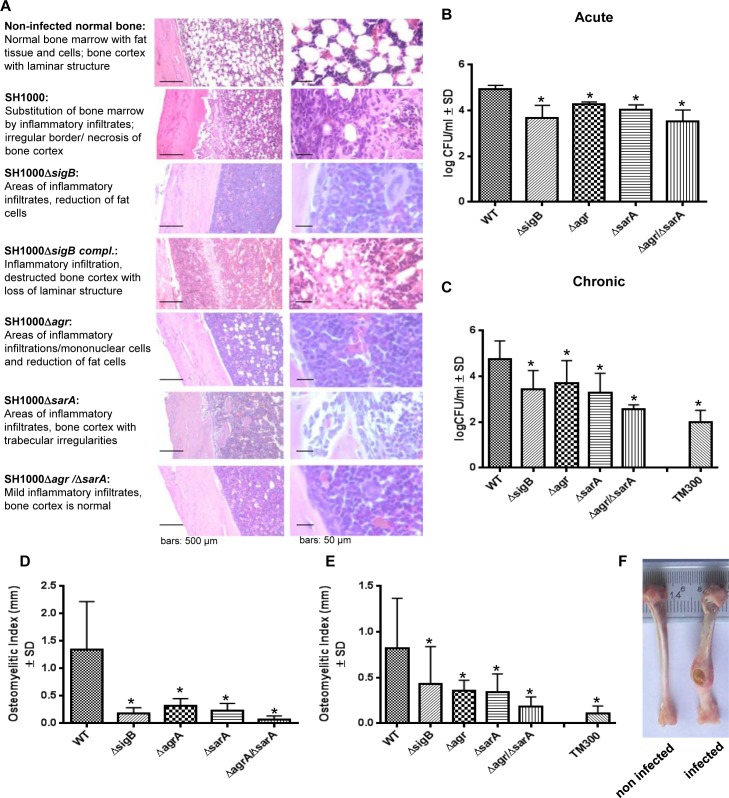
The interplay of *agr*, *sarA* and *sigB* is required to settle and maintain an infection in a local rat chronic osteomyelitis model. A local chronic osteomyelitis model was used to study the effects of wild-type SH1000 and corresponding mutants. (A) After 4 days of infection slices of bone tissue were performed for histology and stained by hematoxylin-eosin to detect the influx of immune cells to bone tissue. For each strain tested representative photomicrographs are shown in low and high magnification and typical histological features are described. (B, C) Bacterial persistence in host tissue was analyzed 4 days p.i. (acute) and 14 weeks p.i. (chronic) by plating homogenized bone tissue on agar plates and counting the CFU the following day. (D, E) The osteomyelitis index was measured 4 days p.i. (acute) and 14 weeks p.i. (chronic) from each infected tibiae in comparison with the non-infected tibiae from the same animal. The experiments were performed with 12 animals per group and the results shown are means ± SD. * P≤0.05 ANOVA test comparing persisting CFU and osteomyelitis index caused by wild-type SH1000 and the corresponding mutants. (F) Photographs of infected (wild-type SH1000) and non-infected tibiae recovered after 14 weeks.

To test the ability of the wild-type and the mutant strains to establish an infection *in vivo*, we next performed experiments using a rat localized osteomyelitis model [41], where defined numbers of bacteria (1x10^6^ CFU; strain SH1000 and mutants) were directly injected into the bones (Fig 6). Using this model we aimed to study the complex interaction of the bacterial strains with the immune response of the host organism. After 4 days (acute stage) and 14 weeks (chronic stage) groups of rats were sacrificed and the tibial bones were used for histology or morphometric analysis (osteomyelitis index; OI, Fig 6F) to assess for infection severity. After 4 days and 14 weeks the bacterial loads were determined by quantitative culture of bone homogenates. The data clearly demonstrated that the single mutants Δ*sigB*, Δ*agr* and Δ*sarA* were found in lower numbers within the bones and caused less inflammation than the wild-type strain (Fig 6B–6E). We further tested as a selective double mutant the *agr*/*sarA* double mutant that persisted in high numbers within cells in culture experiments (Figs 3 and S7). By contrast, in the animal model we detected drastically reduced CFU and a lower osteomyelitis index in rats challenged with the *agr*/*sarA* double mutant when compared with rats infected with the parental wild-type strain. The *agr*/*sarA*-double mutant was found almost as avirulent as the low-pathogenic *Staphylococcus carnosus* strain TM300 [42], which lacks most *S*. *aureus* virulence factors (Fig 6B and 6C). Nevertheless, histological analysis 4 days p.i. revealed that in all experimental groups and control rats the bones were densely infiltrated with immune cells after *S*. *aureus* challenge (Fig 6A), indicating that both the wild-type and the mutant strains attract immune cells and sustain the infection. Only the wild-type strain, however, was able to settle and replicate at high bacterial numbers, to induce severe bone destruction and to develop into chronicity. These findings supports the hypothesis that regulators *agr*, SarA and SigB need to be functional to enable *S*. *aureus* to successfully survive during the whole infection process.

## Discussion

*S*. *aureus* is one of the most frequent causes of osteomyelitis and endovascular infections that often take a therapy-refractory or chronic course. Many clinical studies show that persistent infections are highly associated with the SCV phenotype [4,43]. Only recently, we were able to demonstrate that the formation of dynamic SCVs is an integral part of the long-term infection process that enables the bacteria to hide inside host cells, but also to rapidly revert to the fully-aggressive wild-type phenotype, when leaving the intracellular location and causing a new episode of infection [9]. Therefore, the pathogen-host interaction must be very dynamic and most likely requires global transcriptional changes on the bacterial side to promote survival of the pathogen. This study was aimed at detecting regulatory factors that mediate this dynamic infection and adaptation strategies. To this purpose, we focused on a set of important *S*. *aureus* regulators/regulatory loci *agr*, SarA, and SigB that are linked together in a global regulatory network. Each one of these regulators/regulatory loci is involved in the control of the expression of many virulence factors such as adhesive and cytotoxic components. For our experiments we used various *in vitro* and *in vivo* infection models to analyze the impact and dynamics of the regulators from acute to chronic infection.

In models of acute infection we demonstrated that active *agr-* and SarA-systems are required to cause inflammation and cytotoxicity. Our results are in line with many published infection models, showing that both factors contribute to disease development [27,44–46]. We further confirmed that strains become almost avirulent, when both factors are inactive. In case of a single mutation, *agr* and *sarA* may partly compensate for each other [47], but the compensation is not sufficient in all functions, e.g., in phagosomal escape (Fig 5) and in *in vivo* infections (Fig 6). In the acute stage of infection the bacteria need to express a set of virulence factors, including toxins and exoenzymes, to fight against recruited immune cells [48] and to destroy and invade deep tissue structures at high bacterial numbers. Particularly toxins and cytotoxic factors are under the tight control of *agr* and *sarA* [19,47,49]. The role of SigB in acute infection is less clear. Inactivation of *sigB* in *S*. *aureus* has been reported to decrease infectivity in some murine infection models [50–52], but was ineffective in others [53,54]. In line with these conflicting findings, we observed on the one hand that single mutants in *sigB* express higher levels of toxins, e.g., α-toxin, and become even more virulent (Fig 2C and 2D and S2C and S2D Fig) suggesting a moderating role of *sigB* on the expression of secreted proinflammatory factors. On the other hand double mutants lacking *sigB* were almost avirulent (Fig 2C) indicating a further function of SigB within the regulatory network during acute inflammation. This is supported by recent work showing a fast and transient upregulation of *sigB* in the first hours following host cell invasion and the requirement of SigB for early intracellular growth [55].

In the longer course of the infection bacteria can be situated within host cells, like in the cell culture models. As almost all types of host cells contain killing and clearing machineries [10], the persisting bacteria need to develop mechanisms to resist degradation that can be achieved by two different pathways according to the results from our cell culture experiments:

- Certain mutants that reveal significant metabolic changes including downregulation of virulence factors (Fig 1), and do not escape from the phagosomes after host cell invasion (Fig 5) can persist within their host cells partly at higher numbers than the wild-type phenotype (Fig 3). An example of this is the triple mutant ∆*sigB*∆*agr*∆*sarA* that fails to form SCVs, but is largely avirulent and can persist at high bacterial numbers. Recent work demonstrated that phagosomal escape is largely dependent on the *agr*-regulated phenol-soluble modulins (PSMs) [6,13], but further *agr* or *sarA* regulated factors could also be involved, as single mutants in *agr* or *sarA* were already compromised in their escape [56–58]. This mechanism of persistence is restricted to strains that lack expression of *agr* and/or *sarA*-regulated virulence factors. Our results indicate that these strains are less prone to degradation and can **“passively”** persist inside host cells, possibly within their initial phagosomes after host cell invasion even without forming SCVs.
- Further on, persistence is also possible when bacteria express virulence factors and escape from their phagosomes to the cytoplasm. Persistence obviously requires an adaptation to the intracellular environment that could be attributed to the function of SigB during the long course of the infection. SigB is an important staphylococcal transcription factor that is associated with various types of stress-responses [31,33,35,59], was shown to be upregulated in stable clinical SCVs and was associated with increased intracellular persistence [60]. According to our results SigB is also involved in stress-resistance that promotes **“active”** intracellular survival during long-term persistence: The first important finding is that Δ*sigB*-mutants were not able to persist, as they were completely cleared by their host cells within 9 days (Fig 3B). Additionally, the *agr*/*sarA*-double mutants that were found at the highest numbers during long-term persistence (Figs 3B and S7B) displayed the highest levels of *sigB* expression (Fig 4). Finally, a result of major significance is that SigB is required for dynamic SCV formation, as all mutants deficient in *sigB* were not able to form SCV-phenotypes (Figs 3C and S7C) and *agr*/*sarA*-double mutants that highly expressed *sigB* (Fig 4) developed the highest levels of SCVs. Only recently SigB was described as an important virulence factor in stable SCVs that mediates biofilm formation and promotes intracellular bacterial growth [61]. Yet, in our work the recovered SCVs were not stable, but rapidly reverted back to the wild-type phenotype upon subcultivation. Consequently, we describe the formation of dynamic SCVs for persistence as an additional central function that is dependent on an intact SigB-system.

Taken together, our results demonstrated that intracellular bacterial persistence is promoted by the silencing of *agr*- and *sarA*-regulated factors and/or requires an intact SigB-system. Although strains with deletions in the *agr* and/or *sarA*-system were able to persist at high bacterial numbers in cell culture systems that lack most components of a functioning immune system, they showed severe disadvantages in the *in vivo* model, as they were unable to defend themselves from invading immune cells (Fig 2A and 2B) and were rapidly cleared from the infection focus (Fig 6A and 6B). Consequently, SigB represents a crucial factor to dynamically adapt fully virulent wild-type strains to switch to long-term persistent phenotypes. SigB was described to turn down the *agr* system [36], which is most likely responsible for the enhanced inflammatory activity. The downregulated *agr*-system helps the bacteria to form biofilm [62] and silences aggressiveness for persistence within the host cell [63]. This expression pattern (high *sigB* and low *agr*) of global regulators appears to be characteristic for SCVs and seems to represent a general adaptation response, as it had been described for stable SCVs generated by aminoglycoside treatment as well [35]. Yet, the varying stress conditions encountered by *S*. *aureus* on its way to the intracellular location are less defined and many questions remain to be answered to fully elucidate the complete dynamic bacterial adaption strategies: e.g., (i) which intracellular conditions affect the staphylococcal regulatory factors? (ii) which staphylococcal regulatory factor(s) is/are directly influenced by the intracellular milieu (*agr* or *sigB* or further systems, such as the *mazEF* toxin-antitoxin module [64])? (iii) How are the changes of regulatory factors transferred to an increased SCV formation? (iv) which factors of the SigB regulon are required for persistence [65]? (v) Does SigB increase bacterial resistance against antibiotics? All questions require extensive additional laboratory work to decipher the bacterial adaptation mechanisms in more detail.

In our study we used different bacterial backgrounds and mutants, as well as different *in vitro* and *in vivo* infection models to demonstrate that bacteria apply general adaption strategies via the crosstalk of regulatory factors with a central function for SigB. By this means, bacteria can rapidly react to changing environmental conditions and dynamically adjust their virulence factor expression at any time of the infection. As the regulatory network involving SigB appears to be the central factor that enables the bacteria to persist and cause chronic infections, it represents a novel therapeutic target for prevention and treatment of chronic and recurrent infection.

## Materials and Methods

### Bacterial strains used in the study and generation of bacterial mutants

The bacterial strains and mutants used in this study are listed in Supp. S1 Table. All the experiments were performed with wild type and mutants in the background of *S*. *aureus* LS1 and *S*. *aureus* SH1000. LS1 is a murine arthritis isolate that has been used in infection models before [66]. The strain SH1000 has a complementation of the *rbsU* gene in the strain 8325–4 which is deficient in SigB activity (a stress-induced activity) due to a mutation in the rsbU gene. This gene encodes for a phosphatase required for the release of the sigma factor SigB from inhibition by its anti-sigma factor RsbW. To create a strain with an intact SigB-dependent stress response, the rsbU gene was restored in *S*. *aureus* 8325–4, with the resulting strain called SH1000 [66].The antibiotic resistance cassette-tagged *agr*, *rsbUVWsigB*, and *sarA* mutations of RN6911 [67], IK181 [68] and ALC136 [69] were transduced into LS1 using phages 11, 80α and 85, respectively. Δ*rsbUVWsigB* derivatives were cis-complemented by phage transduction with a resistance cassette-tagged intact *sigB* operon obtained from strain GP268 [70] using phage 80α. The *sarA* mutant of SH1000 was constructed by replacing the *sarA* gene with an erythromycin resistance cassette. The *sigB* mutant of SH1000 was constructed by transducing the *sigB* mutation (*sigB*::*Tn551*) from RUSA168 into SH1000.

### Preparation of protein extracts for proteome analysis of the extracellular protein fraction

The strains were cultivated in tryptic soy broth (TSB) at 37°C with linear shaking at 100 rpm in a water bath (OLS200, Grant Instruments, England). Strains were grown in two sets. In the first set the wild type and the ∆*sigB*, ∆*agr*, ∆*sarA* and ∆*agr*/∆*sarA* mutants were cultured and in a second set the wild type and the ∆*sigB*∆*agr*, ∆*sigB*∆*sarA* and ∆*sigB*∆*agr*∆*sarA* mutants were cultured. The wild type always served as control. During exponential growth phase bacteria were pelleted by centrifugation and culture supernatants were mixed with 10% final concentration of TCA and proteins precipitated at 4°C overnight. Pelleted proteins were washed five times with 70% ethanol and then incubated for 30 min at 21°C and mixed at 600 rpm in a thermomixer (Eppendorf, Germany). Afterwards, pellets were washed once with 100% ethanol and dried in a speed vacuum centrifuge (Concentrator 5301, Eppendorf, Germany). Subsequently, protein pellets were dissolved in a suitable volume of 1x UT buffer (8 M urea and 2 M thiourea) and incubated for 1h at 21°C with shaking at 600 rpm in a thermomixer (Eppendorf, Germany). No soluble components were pelleted via centrifugation. Protein concentration was determined according to Bradford [71]. 4 μg of protein were reduced and alkylated with Dithiothreitol and Iodoacetamid prior to digestion with Trypsin.

### Proteome analysis by mass spectrometry and analysis of data

Peptides were purified and desalted using μC18 ZipTip columns and dried in a speed vacuum centrifuge (Concentrator plus, Eppendorf, Germany). Dried peptides were dissolved in LC buffer A (2% ACN, in water with 0.1% Acetic acid) and subsequently analysis by mass spectrometry was performed on a Proxeon nLC system (Proxeon, Denmark) connected to a LTQ-Orbitrap Velos- mass spectrometer (ThermoElectron, Germany). For LC separation the peptides were enriched on a BioSphere C18 pre-column (NanoSeparations, Netherlands) and separated using an Acclaim PepMap 100 C18 column (Dionex, USA). For separation a 86-minute gradient was used with a solvent mixture of buffer A (2% Acetonitrile in water with 0.1% Acetic acid) and B (ACN with 0.1% acetic acid): 0–2% for 1min, 2–5% for 1 min, 5–25% for 59 min, 25–40% for 10 min, 40–100% for 8 min for buffer B. The peptides were eluted with a flow rate of 300 nL/min. The full scan MS spectra were carried out using a FTMS analyzer with a mass range of m/z 300 to 1700. Data were acquired in profile mode with positive polarity. The method used allowed sequential isolation of the top 20 most intense ions for fragmentation using collision induced dissociation (CID). A minimum of 1000 counts were activated for 10 ms with an activation of q = 0.25, isolation width 2 Da and a normalized collision energy of 35%. The charge state screening and monoisotopic precursor selection was rejecting +1 and +4 charged ions. Target ions already selected for MS/MS were excluded for next 60 seconds.

Analysis of MS data was performed with Rosetta Elucidator version 3.3.0.1 (Rosetta Biosoftware, MA, USA). An experimental definition for differential label free quantification was created with default settings: instrument mass accuracy of 5 ppm, spectral alignment search distance of 4 minutes, peak time width minimum of 0.1. For identification the *S*. *aureus* NCTC 8325 FASTA sequence in combination with SEQUEST/ Sorcerer was used. Oxidation of methionine, carbamidomethylation and zero missed cleavages were specified as variable modifications. After identification an automatic Peptide/Protein Tellers annotation was performed and only Peptide Teller results greater than 0.8 were used. At least two peptides per protein or one peptide with protein sequence coverage of at least 10% were necessary for reliable protein identification.

Protein intensities received with the Rosetta Elucidator software were further processed using Genedata Analyst version 7.6 (Genedata AG, Basel, Switzerland). Protein intensities were normalized using the central tendency normalization with a dynamic target and the median as central tendency. For relative quantification the ratio of the protein intensities between the wild type and the respective mutants from set one or two were calculated. A protein with a ratio of ≥2 was assigned to be upregulated in the wild type and with a ratio of ≤0.5 was assigned to be upregulated in the mutant strain. For prediction of protein localization the PSORT database was used (PSORTdb 3.0, http://db.psort.org/browse/genome?id=9009). Furthermore, for categorizing the proteins into subgroups The SEED (Overbeek et al., Nucleic Acids Res 33(17)) annotation for *S*. *aureus* NCTC8325 was used. A heat map with log_2_ ratios of the wild type versus the respective mutants was created using the package heatmap.plus version 1.3 in R version 2.15.1 (http://www.R-project.org).

### Preparation of bacterial strains and bacterial supernatants

For cell culture experiments, bacteria were grown overnight in 15 ml of brain-heart infusion (BHI) with shaking (160 rpm) at 37°C. The following day, bacteria were two times washed with PBS and were adjusted to OD = 1 (578nm). Bacterial supernatants were prepared as described [21]. Briefly, bacteria were grown in 5 ml of brain-heart-infusion broth (Merck, Germany) in a rotator shaker (200 rpm) at 37°C for 17 h and pelleted for 5 min at 3350 *g*. Supernatants were sterile-filtered through a Millex-GP filter unit (0.22 μm; Millipore, Bedford, MA) and added to the cell culture medium in the indicated concentrations.

### Growth rates and generation time (growth curves)

For growth curves, *Staphylococcus aureus* wild-type LS1, SH1000 and their respective mutants were grown overnight in 15ml of Mueller Hinton infusion (MH) with shaking (160rpm) at 37°C. The following day, 100ml of MH were inoculated with each strain in order to obtain the starting optical density 0,05 (578nm). The growth of each strain was monitored spectrophotometrically (578nm) and by plating on blood agar for counting of CFU every hour during 8h. The growth rate (**μ**, growth speed) and the generation time (**g**) for each strain used in this study were calculated according the followings formulas [72].

$$\begin{array}{l} \frac{µ=\text{LnN}-{\text{LnN}}_{0}}{\text{t}-\text{t}0}\\ \text{g}=\text{Ln}2/µ \end{array}$$

μ = growth rate

g = generation time

N = final population

N0 = initial population

t = final time

t0 = initial time

### Isolation and culture of different cell types and long-term cell culture infection models

Various types of host cells, namely primary isolated human umbilical venous endothelial cells (HUVECs) and primary isolated human osteoblasts were cultivated as outlined before [73–75] and were infected with different *S*. *aureus* strains as previously described [14]. Briefly, primary cells were infected with an MOI (multiplicity of infection) of 50. After 3 h cells were washed and lysostaphin (20 μg/ml) was added for 30 min to lyse all extracellular or adherent staphylococci, then fresh culture medium was added to the cells. The washing, the lysostaphin step and medium exchange was repeated daily to remove all extracellular staphylococci, which might have been released from the infected cells. To detect live intracellular bacteria at different time points post infection (p.i.) host cells were lysed in 3 ml H_2_O (for 25cm^2^ bottles) or 20 ml H_2_O (for 175cm^2^ bottles). To determine the number of colony forming units (CFU), serial dilutions of the cell lysates were plated on blood agar plates and incubated overnight at 37°C. The colony phenotypes were determined on blood agar plates by a Colony Counter Biocount 5000 (Biosys, Karben, Germany).

### Phagosomal escape assays

A549 cells stably expressing the escape marker YFP-CWT in the cytoplasm were generated by transduction with lentiviral particles as described [76]. Transductants were passaged three times and subsequently selected by FACS. The phagosomal escape signal is based on the recruitment of YFP-CWT to the staphylococcal cell wall upon rupture of the phagosomal membrane barrier. Recruitment thereby is mediated by the cell wall targeting domain of the *S*. *simulans* protease lysostaphin [77]. Accumulation of the fusion protein YFP-CWT was recorded by fluorescence microscopy. For this the A549 YFP-CWT cells were infected in imaging dishes with coverglass bottom (MoBiTec, Göttingen, Germany) with the *S*. *aureus* LS1 wild type strain or the corresponding mutants for 1 h, followed by lysostaphin treatment to remove all extracellular bacteria. 2 h post infection the YFP-signal was observed with a Zeiss Axiovert 135 TV microscope (Carl Zeiss, Jena, Germany) equipped with a 50 W HBO mercury short-arc lamp and a Zeiss filter set (excitation BP 450–490 nm, beam splitter FT 510 nm, emission LP 515 nm). For quantitative assays a 100×/NA 1.3 plan-neofluar objective (field of view 25mm) was used. Data were acquired with an AxioCam MRm camera and processed using Zeiss AxioVision software. 10 fields of view were recorded per experiment and phagosomal escape was enumerated for 5 independent experiments per strain.

### Isolation and culture of PMNs

Human polymorphonuclear cells (PMNs) were freshly isolated from Na-citrate-treated blood of healthy donors. For neutrophil-isolation, dextran-sedimentation and density gradient centrifugation using Ficoll-Paque Plus (Amersham Bioscience) was used according to the manufacturer's instruction. Cell purity was determined by Giemsa staining and was always above 99%. PMNs were suspended to a final density of 1×10^6^ cells/0.5 ml in RPMI 1640 culture medium (PAA Laboratories GmbH) supplemented with 10% heat-inactivated FCS (PAA Laboratories GmbH) and immediately used for the experiments. All incubations were performed at 37°C in humidified air with 5% CO_2_.

To measure cell death induction 1x10^6^ cells/0.5 ml PMNs were cultured at 37°C in a 5% CO_2_ atmosphere in 24-well plates and bacterial supernatants were added as indicated. After 1 h cell death induction was analyzed as described previously [78,79].

### Cell death assays in endothelial cells and osteoblasts

For the flow cytometric invasion assay, primary human osteoblasts or endothelial cells were plated at 2x10^5^ cells in 12-well plates the day before the assay. Cells were washed with PBS, then cells were incubated with 1 ml of 1% HSA, 25 mM HEPES (pH 7.4) in F-12 medium (Invitrogen). The bacteria were grown in BHI, washed and the bacterial suspension (OD 1, 540 nm) was prepared and was added to cells. After the lysostaphin step the infected cells were incubated for 24 h and cell death assays were performed by measuring the proportion of hypodiploid nuclei as described [8].

To measure cell death during the bacterial persistence, this protocol was performed every two days.

### Hemolysis assay

Hemolysis analysis was performed as described previously with some modifications [80]. The lysis efficacies of human red blood cells were measured using whole culture supernatants of *S*. *aureus* LS1, SH1000 and their respective mutants. Briefly, *S*. *aureus* cells were cultured in BHI for 17 h at 250rpm. Staphylococcal cells were centrifuged and the supernatants were used for measuring hemolytic activity. Supernatants (100μL) were added to 100μl human red blood cells (previously prepared, see preparation of erythrocytes). To determine hemolytic activities, the mixtures of blood and *S*. *aureus* were incubated at 37°C for 30 minutes. Supernatants were collected by centrifugation at 1000g for 5 min and optical densities were measured at 570nm in an ELISA plate reader. The strain *S*. *aureus* Wood46 was used as a positive control.

#### Preparation of erythrocytes

A volume of 32ml blood was mixed with 8ml sodium citrate; 20ml Dextran was added and the mix was incubated for 45–60 minutes to form the gradient sedimentation. The white blood cells were removed from the top layer and the erythrocytes were prepared in a 1.4% solution in PBS. In the mixture with staphylococcal supernatant, the final concentration was approximately 0.7% v/v.

These results and the description of the method was added in the supporting information of our manuscript (S1C Fig).

### Measurement of cytokines release

For measurement of cytokine release, osteoblasts were seeded in 12-well plates and stimulated with live staphylococci as described above. After the lysostaphin step, cells were washed and incubated with new culture medium for 24 h. Conditioned media were centrifuged to remove cells and cellular debris, and samples were frozen at 20°C until levels of cytokines and chemokines were measured. The levels of RANTES [regulated on activation of normal T cell expressed and secreted] was measured using immunoassays from RayBiotech according to the manufacturer`s description. Results are given as absolute values (ng/ml) of two independent experiments performed in duplicates. Statistical analysis was performed using the ANOVA test.

### Rat chronic osteomyelitis model

Outbred Wistar adult rats (groups of 10–12 rats) weighing 250–350 g were anesthetized with ketamine/xylazine. The left tibia was exposed and a hole made with a high-speed drill using a 0.4 mm diameter bit. Each tibia was injected with a 5 μl suspension containing 1x10^6^ CFU of bacteria suspended in fibrin glue (Tissucol kit 1 ml, Baxter Argentina-AG Viena, Austria). Groups of rats were sacrificed at 4 days or 14 weeks after intratibial challenge by exposure to CO_2_. Both left and right tibias were removed and morphometrically assessed by measuring the Osteomyelitic Index (OI) as described previously [41]. Both epiphysis were cut off from the infected tibias and 1 cm bone segments involving the infected zone were crushed and homogenized. Homogenates were quantitatively cultured overnight on trypticase soy agar and the number of CFU was determined. The OI and the *S*. *aureus* CFU counts from each experimental group (infected and control) were compared.

### Extraction of RNA and real-time PCR

For RNA extraction, we used the kit RNeasy Mini kit (Qiagen). The RNA extraction was performed following the manufacture instruction and the suggestions of the protocol described by Garzoni et al. [81].

All the primers used in this study are listed in Supp. S2 Table. Real-time PCR was performed by using the RNA isolated from infected cells, infected tissues or different bacterial isolates. The cDNA was obtained using the kit QuantiTect reverse transcription (Qiagen) and iQSYBR Green Supermix (BIO-RAD) was used. The reaction mixtures were incubated for 15 min at 95°C followed by 40 cycles of 15 s at 95°C, 30 s at 55°C and 30 s at 72°C using the iCycler from BIO-RAD. PCR efficiencies, melting-curve analysis and expression rates were calculated with the BIO-RAD iQ5 Software.

In order to analyze the expression of bacterial and host cell genes, respectively, the primers listed in S2 Table were used [14,63]. The expression analysis experiments were performed using the software CFX Manager software which calculates the normalized expression ΔΔC_T_ (relative quantity of genes of interest is normalized to relative quantity of the reference genes across samples). The genes used as housekeeping genes to analyze the chemokine expression were GAPDH and B-actin. As controls we used uninfected host cells. For bacterial factors, we used as housekeeping genes *aroE*, *gyrB* and *gmk*. The results shown in each graph are normalized to that control (control expression = 1).

### Ethics statement

The isolation of human cells and the infection with clinical strains were approved by the local ethics committee (Ethik-Kommission der Ärztekammer Westfalen-Lippe und der Medizinischen Fakultät der Westfälischen Wilhelms-Universität Münster). For our study, written informed consent was obtained (Az. 2010-155-f-S). Taking of blood samples from humans and animals and cell isolation were conducted with approval of the local ethics committee (2008-034-f-S; Ethik-Komission der Ärztekammer Westfalen-Lippe und der Medizinischen Fakultät der Westfälischen Wilhelms-Universität Münster). Human blood samples were taken from healthy blood donors, who provided written informed consent for the collection of samples and subsequent neutrophil isolation and analysis.

For the local osteomyelitis model care of the rats was in accordance with the guidelines set forth by the National Institutes of Health [82]. The experimental protocol involving rats in the experiments was approved by the Institutional Committee for the Care and Use of Laboratory Animals (CICUAL), School of Medicine, University of Buenos Aires (resolution CD 2361–11).

### Statistical analysis

The relationship between WT and all the different mutants was established by the one way ANOVA test with the Dunnett multi-comparison post-test. Significance was calculated using the GraphPad Prism 6.0 software and results were considered significant at P = 0.05.

## Supporting Information

S1 Table Strains used in this study. (DOCX)

S2 Table Primers used in this study. (PPTX)

S3 Table Summary of proteins identified by LC-MS/MS in the secretome of LS1 and its isogenic mutant derivatives and analysis of the impact of inactivation of SarA on protein levels in the secretome in comparison to expression/ protein levels in previously reported transcriptome and proteome studies. (DOCX)

S1 Fig Growth curves, mean generation times (MTGs) and hemolytic activities of bacterial strains. The growth curves of the wild-type strains LS1 (A) and SH1000 (B) and the corresponding mutants were performed in Müller-Hinton medium in order to determinate the Mean Generation Times (g) and growing rates (μ) (table; according the description in materials and methods). All the generation times of the different strains were not significantly different (ANOVA test, P>0.05). (C) The degree of hemolysis was evaluated in all the strains used in our study by measuring hemolytic activity spectrophotometrically to detect the release of hemoglobin from freshly isolated red blood cells (OD:570nm). *S*. *aureus* Wood 46 was used as a positive control. All the results are given relative to the values of *S*. *aureus* Wood 46 (hemolysis = 1). The graph represents the means of four independent experiments ±SD. All the groups were compared against the wild-type strains by ANOVA test, * P≤0.05.
(PPTX)

S2 Fig Cytotoxic effect of SH1000, LS1 and mutants on PMNs. Cytotoxicity experiments were performed in polymorphonuclear cells (PMNs) using wild-type strains LS1, SH1000 and their derivate mutants. (A, B; infected with SH1000 and their derivate mutants) PMNs were freshly isolated from human blood (A) and bone marrow of Balb/C mice (B) and 1×10^6^/0.5 ml cells were incubated with 50% v/v of bacterial supernatants for 1 h. Then cells were washed, stained with annexin V and propidium iodide and cell death was measured by flow cytometry. (C, D) PMNs were freshly isolated form human blood and 1×10^6^/0.5 ml cells were incubated with different % v/v (C) or 2,5% v/v of bacterial supernatants of LS1, LS1ΔsigB and LS1ΔsigB compl. for 1 h (D). Then cells were washed, stained with annexin V and propidium iodide and cell death was measured by flow cytometry. The values of all experiments represent the means ± SD of at least three independent experiments. * P≤0.05 ANOVA test was used to compare the effects induced by the wild-type strains and the corresponding mutants.
(PPTX)

S3 Fig Inflammatory effects of SH1000 and mutants on osteoblasts. The inflammatory effects of LS1, SH1000 and their mutants were evaluated on human osteoblasts. (A, B) Cultured osteoblasts were infected with LS1, SH1000 or their derivate mutants (MOI 50). After bacterial invasion (3 h) extracellular staphylococci were removed and infected cells were incubated with culture medium for 48 h. To analyze host cell response the changes in the expression of the chemokine CXCL-11 and CCL-5 were measured by real-time PCR. Results demonstrate the relative increase in gene expression, compared to unstimulated cells (control = 1). The values of all experiments represent the means ± SD of at least three independent experiments. (C, D) Measurement of chemokine release in cell culture supernatants by enzyme-linked immunosorbent assay (ELISA). Confluent human primary osteoblasts were infected with live LS1, SH1000 and their respective mutants (multiplicity of infection, 50) and incubated for 24 h as described in Materials and Methods. The conditioned media were analyzed for RANTES (regulated on activation of normal T cell expressed and secreted). Results are means ± SD of 2 independent experiments performed in duplicates. * P≤0.05 ANOVA test was used to compare the effects induced by the wild-type strains and the corresponding mutants.
(PPTX)

S4 Fig Comparison of bacteria uptake and cell activation between osteoblasts and endothelial. (A) The uptake of *S*. *aureus* wild-type strains LS1 and SH1000 were measured in human endothelial cells (HUVECs) and osteoblasts by plating cell lysates directly after infection and counting the CFU on the following day. (B) The cell activation of *S*. *aureus* wild-type strains LS1 and SH1000 were measure in osteoblasts and endothelial cells (HUVECs) by real time after 48h post infection. The values represent the means ± SD of three independent experiments performed in triplicate. * P≤0.05 t-test comparing the two cell types. (C, D) Cultured HUVECs were infected with *S*. *aureus* SH1000 or their derivate mutants (MOI 50). After bacterial invasion (3 h) extracellular staphylococci were removed by washing and lysostaphin treatment and infected cells were incubated with culture medium for 48 h. To analyze host cell response the changes in the expression of the chemokine CXCL-11 were measured by real-time PCR. Results demonstrate the relative increase in gene expression, compared to unstimulated cells (control = 1). The values of all experiments represent the means ± SD of at least three independent experiments. * P≤0.05 ANOVA test was used to compare the effects induced by the wild-type strains and the corresponding mutants.
(PPTX)

S5 Fig Host cell invasion after infection of osteoblasts with LS1 and SH1000 and their derivate mutants. The initial intracellular counts of *S*. *aureus* wild-type strains LS1 and SH1000 and their respective mutants were measured in human osteoblasts by plating cell lysates directly after infection (time zero). The values represent the means ± SD of three independent experiments performed in triplicate. No significant differences were found between the different strains (ANOVA p>0,05).
(PPTX)

S6 Fig Cytotoxicity during bacterial persistence in osteoblasts. (A) Osteoblasts were infected with LS1 (WT) and mutants as described before. After the lysostaphin step the infected cells were incubated with medium and cell death assays were performed by measuring the proportion of hypodiploid nuclei as described. These measurements were performed every 2 days after infection. (B, C) The rates of cell death after 1 and 9 days post infection is shown separately. The values of all experiments represent the means ± SD of at least three independent experiments. * P≤0.05 ANOVA test was used to compare the effects induced by the wild-type strain and the corresponding mutants.
(PPTX)

S7 Fig Infection of osteoblasts and endothelial cells with SH1000 and mutants over 9 days. (A) Cultured osteoblasts were infected with *S*. *aureus* strain SH1000 or the corresponding or complemented mutants as described and infected cells were analysed for up to 9 days. The numbers of viable intracellular persisting bacteria were determined every 2 days by lysing host cells, plating the lysates on agar plates and counting the colonies that have grown on the following day. (B) The results after 9 days are demonstrated separately. The results shown here are from osteoblast infection experiments, but similar results were obtained with endothelial cells. (C) The percentage of small and very small (SCV) phenotypes (<5 and <10-fold smaller than those of the wild-type phenotypes, respectively) recovered (between 200 and 500 colonies examined in each sample) were determined after 7 days p.i. The values of all experiments represent the means ± SD of at least three independent experiments. * P≤0.05 ANOVA test comparing the effects induced by the wild-type strain and the corresponding mutants. (D) Photographs of recovered colonies were performed after 7 days of infection of endothelial cells with strains LS1, LS1∆sigB or LS1∆sigBcompl.
(PPTX)

S8 Fig Infection of osteoblasts with LS1 and mutants for *sae* or *hla* over 9 days. Cultured osteoblasts were infected with *S*. *aureus* wild-type strain LS1 or the corresponding mutants for *sae* (A, B) or *hla* (C, D) as described and infected cells were analysed for up to 9 days. (A, C) The numbers of viable intracellular persisting bacteria were determined every 2 days by lysing host cells, plating the lysates on agar plates and counting the colonies that have grown on the following day. The values represent the means ± SD of at least three independent experiments. *P≤0.05; T-test comparing the effects induced by the wild-type strain and the corresponding mutants did not reveal significant differences at any time point measured. (B, D) The cell death was monitored at each time point by FACS analysis. The values represent the means ± SD of at least three independent experiments. ANOVA was used to compare at different time points the effects of the wild-type strain and mutants on cell death induction in relation to control cells. * P≤0.05. (E) The cytokine release for LS1 (WT) and the corresponding mutants Δ*sae* and Δ*hla* was analyzed in primary osteoblast by ELISA as described in materials and methods. The differences between WT and mutants were not significant (ANOVA p>0,05).
(PPTX)
