# Supplementary material for: Sigma Factor SigB Is Crucial to Mediate Staphylococcus aureus Adaptation during Chronic Infections
Source: PLoS Pathog. 2015 Apr 29;11(4):e1004870. doi: 10.1371/journal.ppat.1004870 (PMC4414502; doi:10.1371/journal.ppat.1004870)
Supplement: S1 Table — (DOCX) [file ppat.1004870.s001.docx]

| Strain name in the paper | Strain | Description | Source/ Reference |
| --- | --- | --- | --- |
| LS1 | LS1 | septic arthritis isolate | [1] |
| LS1Δ*sigB* | BB1591 | LS1 derivative carrying the *rsbUVWsigB* deletion of IK181; Em^R^ | [2] |
| LS1Δ*sigB* compl. | HOM300 | BB1591 derivative, *cis*-complemented with a functional *sigB* operon of GP268; Tc^R^ | This work |
| LS1Δ*agr* | HOM150 | LS1 derivative carrying the *agr::tetM* deletion of RN6911; Tc^R^ | This work |
| LS1Δ*sarA* | HOM174 | LS1 derivative carrying the *sar::*Tn*917*LTV1 mutation of ALC136; Em^R^ | This work |
| LS1 Δ*agr*/ Δ*sarA* | HOM175 | LS1 derivative carrying the *agr::tetM* and *sar::*Tn*917*LTV1 mutations of RN6911 and ALC136, respectively; Em^R^, Tc^R^ | This work |
| LS1 Δ*sigB*/Δ*agr* | HOM297 | LS1 derivative carrying the *rsbUVWsigB::ermB* and *agr::tetM* mutations of IK181 and RN6911, respectively; Em^R^, Tc^R^ | This work |
| LS1 Δ*sigB*/Δ*sarA* | HOM298 | LS1 derivative carrying the *rsbUVWsigB::ermB* and *sarA::*Km mutations of IK181 and PC1839, respectively; Em^R^, Km^R^ | This work |
| LS1 Δ*sigB*/ Δ*agr*/Δ*sarA* | HOM299 | LS1 derivative carrying the *rsbUVWsigB::ermB, agr::tetM,* and *sarA::*Km mutations of IK181, RN6911, and PC1839, respectively; Em^R^, Km^R^, Tc^R^ | This work |
| SH1000 | SH1000 | 8325-4 with functional *rsbU* | [3] |
| SH1000 Δ*sigB* | HOM184 | SH1000 derivative carrying the *rsbUVWsigB* deletion of IK181; Em^R^ | This work |
| SH1000 Δ*sigB* compl. | HOM301 | HOM184 derivative, *cis*-complemented with a functional *sigB* operon of GP268; Tc^R^ | This work |
| SH1000 Δ*agr* | SH1001 | SH1000 derivative carrying the *agr::tet* mutation of RN6911; Tc^R^ | [3] |
| SH1000 Δ*sarA* | SH1002 | SH1000 derivative carrying the *sarA::*Km mutation of PC1839; Km^R^ | [3] |
| SH1000 Δ*agr*/Δ*sarA* | ALC2963 | SH1000 derivative carrying the *agr*::*tetM* and *sarA::*Km mutations of RN6911 and PC1839, respectively; Tc^R^, Km^R^ | A. Cheung, unpublished |
| LS1 Δ*hla* | LS1 Δ*hla* | LS1 Δ*hla*, Erm^R^, obtained by phage transduction from DU1090 | [4] |
| LS1 Δ*sae* | LS1 Δ*sae* | LS1 Δ*sae*, Kan^R^,obtained by phage transduction from New 29 | [4] |
| ALC136 | ALC136 | RN6390 *sar::*Tn*917*LTV1 transposon mutant; Em^R^ | [5] |
| GP268 | GP268 | BB255 derivative with a functional *rsbU*; Tc^R^ | [6] |
| IK181 | IK181 | BB255 derivative carrying a *rsbUVWsigB::ermB* deletion; Em^R^ | [7] |
| PC1839 | PC1839 | 8325-4 derivative with a *sarA::*Km insertional mutation; Km^R^ | [8] |
| RN6911 | RN6911 | RN6390 derivative carrying a *agr::tetM* deletion; Tc^R^ | [9] |
| DU 1090 | DU 1090 | 8325-4, *hla-erm1*, pTS01, Erm^R^, Tet^R^ | [10] |
| New 29 | New 29 | Newman Δ*saePQRS,* Kan^R^ | [11] |
| Wood46 | Wood 46 | *S. aureus* wood46 | [12] |

Reference List

1. Ahmed S, Meghji S, Williams RJ, Henderson B, Brock JH et al. (2001) *Staphylococcus aureus* fibronectin binding proteins are essential for internalization by osteoblasts but do not account for differences in intracellular levels of bacteria. Infect Immun 69: 2872-2877.

2. Nair SP, Bischoff M, Senn MM, Berger-Bachi B (2003) The sigma B regulon influences internalization of Staphylococcus aureus by osteoblasts. Infect Immun 71: 4167-70.

3. Horsburgh MJ, Aish JL, White IJ, Shaw L, Lithgow JK et al. (2002) *sigmaB* modulates virulence determinant expression and stress resistance: characterization of a functional rsbU strain derived from *Staphylococcus aureus* 8325-4. J Bacteriol 184: 5457-5467.

4. Schmitt J, Joost I, Skaar EP, Herrmann M, Bischoff M (2012) Haemin represses the haemolytic activity of Staphylococcus aureus in an Sae-dependent manner. Microbiology 158: 2619-2631.

5. Booth MC, Cheung AL, Hatter KL, Jett BD, Callegan MC et al. (1997) Staphylococcal accessory regulator (sar) in conjunction with agrA contributes to Staphylococcus aureus virulence in endophthalmitis. Infect Immun 65: 1550-1556.

6. Giachino P, Engelmann S, Bischoff M (2001) Sigma(B) activity depends on RsbU in *Staphylococcus aureus*. J Bacteriol 183: 1843-1852.

7. Kullik I, Giachino P, Fuchs T (1998) Deletion of the alternative sigma factor sigmaB in Staphylococcus aureus reveals its function as a global regulator of virulence genes. J Bacteriol 180: 4814-20.

8. Chan PF, Foster SJ (1998) Role of SarA in virulence determinant production and environmental signal transduction in *Staphylococcus aureus*. J Bacteriol 180: 6232-6241.

9. Novick RP, Ross HF, Projan SJ, Kornblum J, Kreiswirth B et al. (1993) Synthesis of staphylococcal virulence factors is controlled by a regulatory RNA molecule. EMBO J 12: 3967-3975.

10. O'Reilly M, de Azavedo JC, Kennedy S, Foster TJ (1986) Inactivation of the alpha-haemolysin gene of Staphylococcus aureus 8325-4 by site-directed mutagenesis and studies on the expression of its haemolysins. Microb Pathog 1: 125-38.

11. Geiger T, Goerke C, Mainiero M, Kraus D, Wolz C (2008) The virulence regulator Sae of Staphylococcus aureus: promoter activities and response to phagocytosis-related signals. J Bacteriol 190: 3419-3428.

12. Abbas-Ali B, Coleman G. Nutritional shifts and their effect on the secretion of extracellular proteins by Staphylococcus aureus (Wood 46). Biochem Soc Trans. 1977;5(2):420-2.
