# Supplementary figures and images for: Sigma Factor SigB Is Crucial to Mediate Staphylococcus aureus Adaptation during Chronic Infections
Source: PLoS Pathog. 2015 Apr 29;11(4):e1004870. doi: 10.1371/journal.ppat.1004870 (PMC4414502; doi:10.1371/journal.ppat.1004870)

## Slide 1
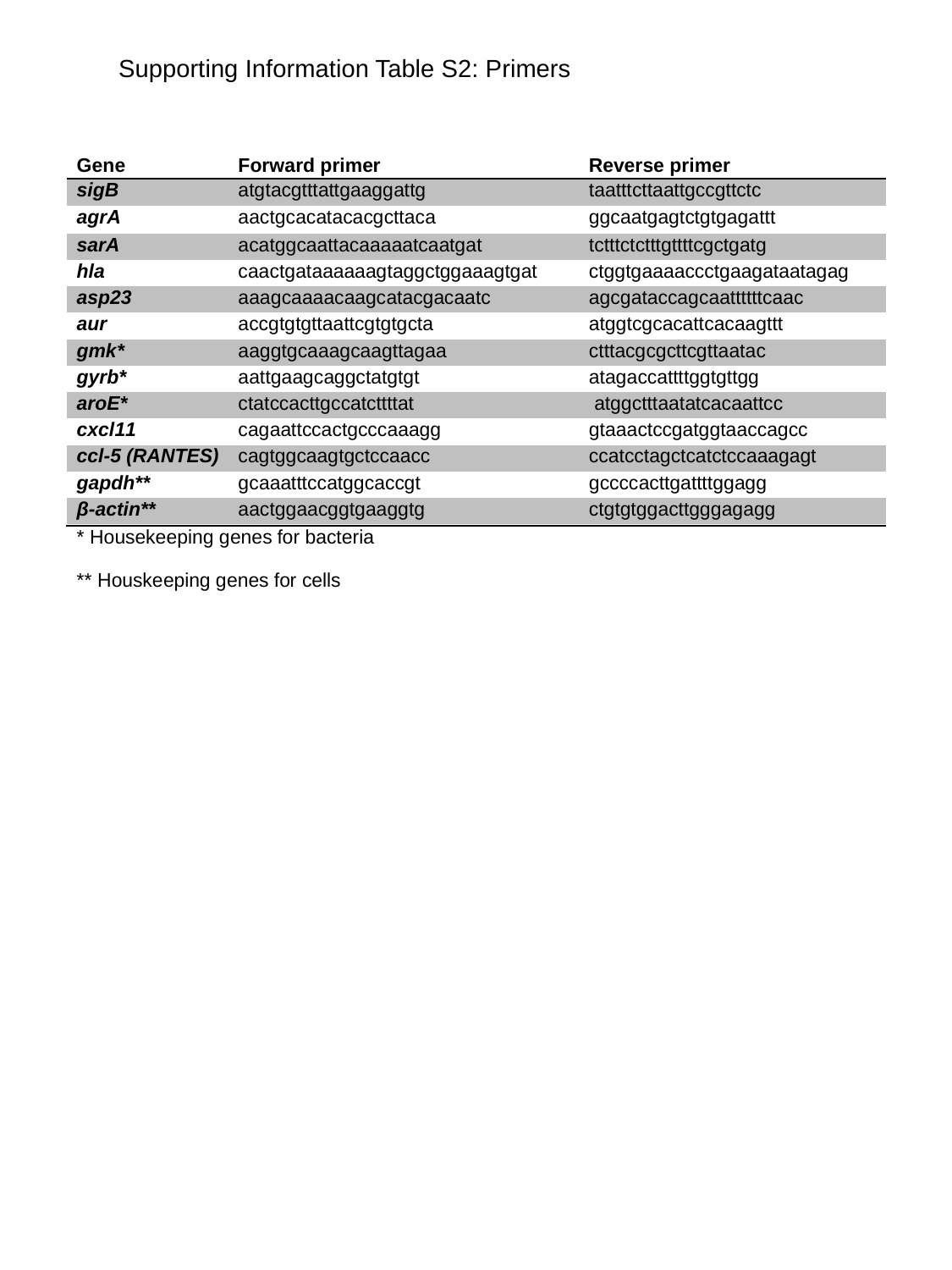

Supporting Information Table S2: Primers

Supplement: S2 Table — (PPTX) [file ppat.1004870.s002.pptx]

## Slide 1
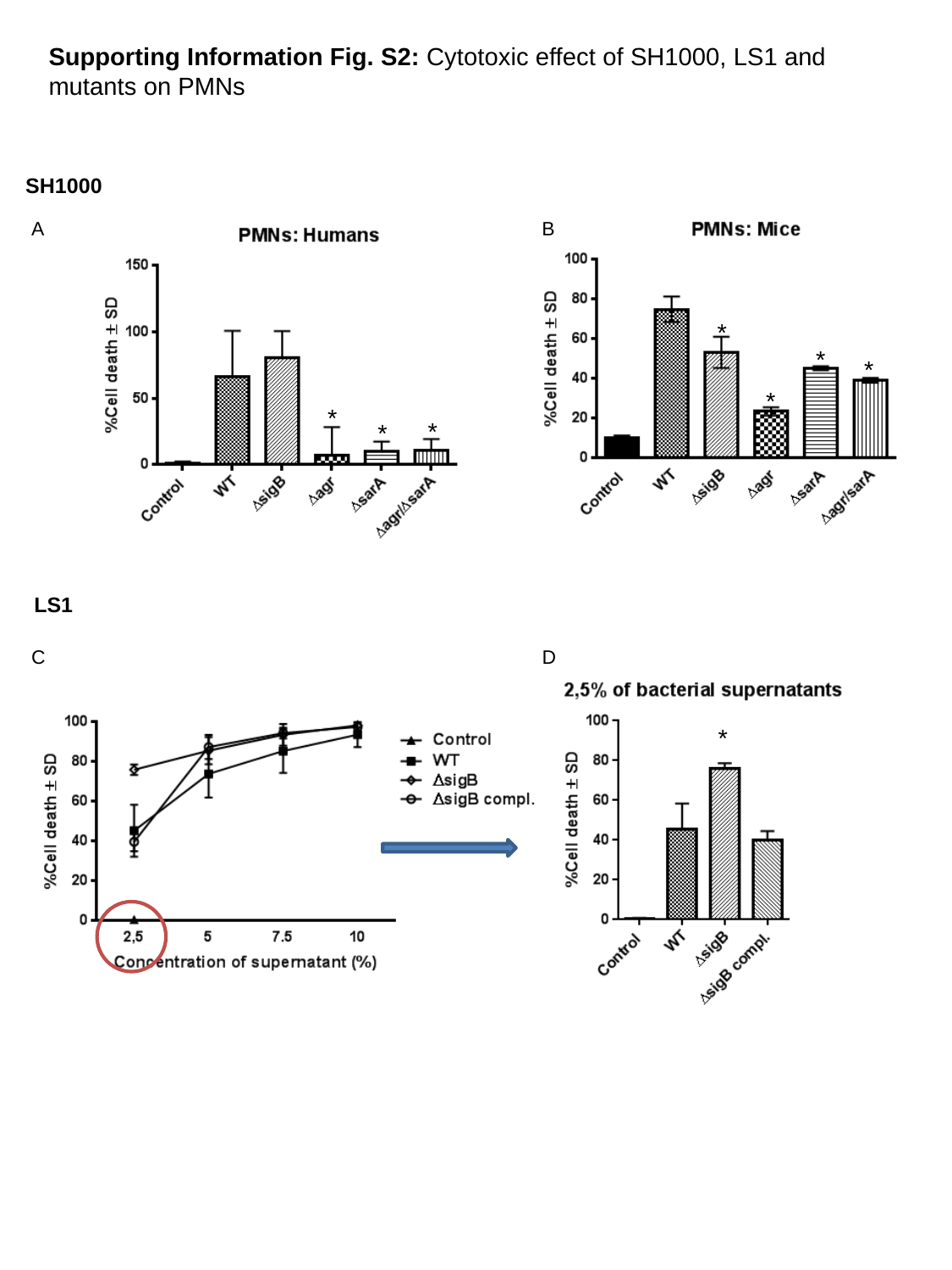

Supporting Information Fig. S2: Cytotoxic effect of SH1000, LS1 and mutants on PMNs
SH1000
A
B
*
*
*
*
*
*
*
LS1
D
C
*

Supplement: S2 Fig — Cytotoxicity experiments were performed in polymorphonuclear cells (PMNs) using wild-type strains LS1, SH1000 and their derivate mutants. (A, B; infected with SH1000 and their derivate mutants) PMNs were freshly isolated from human blood (A) and bone marrow of Balb/C mice (B) and 1×106/0.5 ml cells were incubated with 50% v/v of bacterial supernatants for 1 h. Then cells were washed, stained with annexin V and propidium iodide and cell death was measured by flow cytometry. (C, D) PMNs were freshly isolated form human blood and 1×106/0.5 ml cells were incubated with different % v/v (C) or 2,5% v/v of bacterial supernatants of LS1, LS1ΔsigB and LS1ΔsigB compl. for 1 h (D). Then cells were washed, stained with annexin V and propidium iodide and cell death was measured by flow cytometry. The values of all experiments represent the means ± SD of at least three independent experiments. * P≤0.05 ANOVA test was used to compare the effects induced by the wild-type strains and the corresponding mutants. (PPTX) [file ppat.1004870.s005.pptx]
