# Supplementary material for: Sigma Factor SigB Is Crucial to Mediate Staphylococcus aureus Adaptation during Chronic Infections
Source: PLoS Pathog. 2015 Apr 29;11(4):e1004870. doi: 10.1371/journal.ppat.1004870 (PMC4414502; doi:10.1371/journal.ppat.1004870)
Supplement: S3 Table — (DOCX) [file ppat.1004870.s003.docx]

**Table S3: Summary of proteins identified by LC-MS/MS in the secretome of LS1 and its isogenic mutant derivatives and analysis of the impact of inactivation of SarA on protein levels in the secretome in comparison to expression/ protein levels in previously reported transcriptome and proteome studies**

The list of proteins covered in our secretome analysis was compared with previous transcriptional profiling [1] and proteome studies which analyzed the impact of SarA [2,3]. Levels of many of the proteins covered in our secretome study were influenced by SarA and a summary of the comparison with the literature data and the impact of a knockout of *sarA* in our study is provided in the supplementary table 3 below. Proteins, which were described by Dunman et al., 2001, Jones et al., 2008 and/or Zielinska et al., 2012 as influenced in their expression level by SarA, are marked bold.

This table illustrates the complex influence of SarA onto the secretome, which includes changes in the levels of many proteins. In most cases the directions of change match those observed in literature. Differences might originate from variations in strain background, which have been noted to influence complex gene regulation in *S. aureus* before.

| **Locus tag^1^** | **Protein name** | **Pan gene symbol** | **Intensity ratio LS1 wt / ∆*sarA* (this study)** | **Effect of SarA on expression of encoding genes (Dunman *et al*., 2001)** | **Effect of SarA on protein levels** | |  |
| --- | --- | --- | --- | --- | --- | --- | --- |
|  |  |  |  |  | **Jones *et al*., 2008** | **Zielinska *et al*., 2012** |  |
| **Virulence, Disease and Defence** | | | | | | | |
| **SAOUHSC_00812** | **ClfA** | **clfA** |  |  | **-^2^** | **+** |  |
| **SAOUHSC_02963** | **ClfB** | **clfB** |  |  | **-** | **+** |  |
| **SAOUHSC_00192** | **Coa** | **coa** | **+** |  | **+** |  |  |
| **SAOUHSC_01501** | **EbpS** | **ebpS** | **+** |  | **+** | **+** |  |
| **SAOUHSC_01110** | **Efb** | **efb** | **+** |  | **+** | **+** |  |
| SAOUHSC_00816 | Emp/ Ssp | ssp | + |  |  |  |  |
| SAOUHSC_02630 | EmrA | emrA |  |  |  |  |  |
| SAOUHSC_01114 | Fib | efb | + |  |  |  |  |
| SAOUHSC_02404 | FmtB | fmtB |  |  |  |  |  |
| **SAOUHSC_02803** | **FnBPA** | **fnbA** |  | **+** | **-** |  |  |
| **SAOUHSC_02802** | **FnBPB** | **fnbB** |  | **+** |  |  |  |
| SAOUHSC_00814 | GraB | graB | + |  |  |  |  |
| **SAOUHSC_02710** | **HlgB** | **hlgB** |  | **+** | **+** | **-** |  |
| **SAOUHSC_02709** | **HlgC** | **hlgC** | **+** | **+** |  |  |  |
| **SAOUHSC_01121** | **Hla** | **hla** | **+** | **+** |  | **+** |  |
| **SAOUHSC_01954** | **LukD** | **lukD** | **-** |  |  | **-** |  |
| SAOUHSC_01955 | LukE | lukE | - |  |  |  |  |
| SAOUHSC_01739 | LytH | lytH | + |  |  |  |  |
| SAOUHSC_01873 | Mrp/SasC | mrp |  |  |  |  |  |
| SAOUHSC_00094 | SasD | sasD |  |  |  |  |  |
| SAOUHSC_02982 | SasF | sasF | + |  |  |  |  |
| SAOUHSC_02798 | SasG |  | + |  |  |  |  |
| **SAOUHSC_02706** | **Sbi** | **sbi** | **+** |  | **+** | **+** |  |
| **SAOUHSC_00545** | **SdrD** | **sdrD** |  |  |  | **+** |  |
| **SAOUHSC_00069** | **Spa** | **spa** | **+** | **-** | **+** |  |  |
| SAOUHSC_02161 | truncated MapW | truncated mapW | + |  |  |  |  |
| SAOUHSC_00668 | VraG | vraG |  |  |  |  |  |
| SAOUHSC_00817 | vWbp | vwb |  |  |  |  |  |
|  |  |  |  |  |  |  |  |
| **Cell Wall and Capsule** | |  |  |  |  |  |  |
| **SAOUHSC_00994** | **Atl** | **atl** | **+** | **-** | **-** | **+** |  |
| **SAOUHSC_01081** | **IsdA** | **isdA** |  |  | **+** |  |  |
| **SAOUHSC_00225** | **IspD2** | **ispD** |  |  |  | **+** |  |
| **SAOUHSC_00728** | **LtaSA** | **ltaS** | **+** |  |  | **+** |  |
| SAOUHSC_01467 | Pbp2 | pbp2 |  |  |  |  |  |
|  |  |  |  |  |  |  |  |
| **Metabolism** |  |  |  |  |  |  |  |
| SAOUHSC_01492 | EngA | engA |  |  |  |  |  |
| **SAOUHSC_01207** | **Ffh** | **ffh** |  |  |  | **+** |  |
| SAOUHSC_00248 | LytM | lytM | + |  |  |  |  |
| **SAOUHSC_01360** | **MsrA1** | **msrA** |  |  |  | **+** |  |
| **SAOUHSC_01432** | **MsrA2** | **msrA** |  |  |  | **+** |  |
| SAOUHSC_01621 | NusB | nusB |  |  |  |  |  |
| SAOUHSC_01262 | RecA | recA |  |  |  |  |  |
| SAOUHSC_02511 | RplD | rplD |  |  |  |  |  |
| **SAOUHSC_02478** | **RplM** | **rplM** | **-** |  |  | **+** |  |
| **SAOUHSC_02492** | **RplO** | **rplO** | **-** |  |  | **+** |  |
| **SAOUHSC_01757** | **RplU** | **rplU** |  |  |  | **+** |  |
| **SAOUHSC_02510** | **RplW** | **rplW** | **-** |  |  | **+** |  |
| **SAOUHSC_00524** | **RpoB** | **rpoB** | **+** |  |  | **+** |  |
| **SAOUHSC_01177** | **RpoZ** | **rpoZ** |  |  |  | **+** |  |
| SAOUHSC_00749 | SstD | sstD |  |  |  |  |  |
| SAOUHSC_01764 | ComC | comC |  |  |  |  |  |
| SAOUHSC_02699 | TcyA | tcyA | + |  |  |  |  |
| **SAOUHSC_00074** | **SirA** | **sirA** | **+** |  |  | **+** |  |
| **SAOUHSC_00988** | **SspA** | **sspA** |  |  | **-** | **-** |  |
| **SAOUHSC_01822** | **Tpx** | **tpx** | **-** |  |  | **+** |  |
| SAOUHSC_01058 | TypA | typA |  |  |  |  |  |
| SAOUHSC_00444 |  |  | - |  |  |  |  |
| SAOUHSC_01741 | DtD | dtd |  |  |  |  |  |
|  |  |  |  |  |  |  |  |
| **Stress Response** |  |  |  |  |  |  |  |
| SAOUHSC_01282 | BsaA | bsaA | + |  |  |  |  |
| SAOUHSC_01688 | LepA | lepA |  |  |  |  |  |
| SAOUHSC_01030 |  |  | - |  |  |  |  |
| SAOUHSC_01814 | UspA_2 | uspA_2 |  |  |  |  |  |
| SAOUHSC_01874 |  |  |  |  |  |  |  |
| **SAOUHSC_01653** | **SodA** | **sodA** | **-** |  |  | **+** |  |
| SAOUHSC_00093 | SodM | sodM | - |  |  |  |  |
| SAOUHSC_02133 | PncB | pncB | + |  |  |  |  |
|  |  |  |  |  |  |  |  |
| **Others** |  |  |  |  |  |  |  |
| **SAOUHSC_00364** | **AhpF** | **ahpF** |  |  |  | **+** |  |
| **SAOUHSC_02964** | **ArcR** | **arcR** | **+** | **-** |  |  |  |
| **SAOUHSC_02341** | **AtpD** | **atpD** | **-** |  |  | **+** |  |
| SAOUHSC_02343 | AtpG | atpG |  |  |  |  |  |
| SAOUHSC_02346 | AtpH | atpH | - |  |  |  |  |
| **SAOUHSC_02971** | **Aur** | **aur** | **-** | **-** | **-** | **-** |  |
| **SAOUHSC_02849** | **CidC** | **cidC** |  |  |  | **+** |  |
| SAOUHSC_01496 | Cmk | cmk |  |  |  |  |  |
| SAOUHSC_00258 | EsaA | esaA | - |  |  |  |  |
| SAOUHSC_00921 | Fab | fab |  |  |  |  |  |
| SAOUHSC_00947 | FabI | fabI | - |  |  |  |  |
| SAOUHSC_02926 | FdaB | fdaB | - |  |  |  |  |
| **SAOUHSC_00708** | **FruA** | **fruA** | **+** |  |  | **-** |  |
| SAOUHSC_00707 | FruB | fruB | - |  |  |  |  |
| **SAOUHSC_01205** | **FtsY** | **ftsY** | **+** |  |  | **+** |  |
| SAOUHSC_00300 | Geh | geh |  |  | + |  |  |
| **SAOUHSC_00897** | **GlpQ** | **glpQ** |  |  |  | **+** |  |
| **SAOUHSC_02703** | **GpmA** | **gpmA** |  |  |  | **+** |  |
| SAOUHSC_02000 | GsaB | gsaB |  |  |  |  |  |
| SAOUHSC_00733 | HisC | hisC | + |  |  |  |  |
| SAOUHSC_01968 | Hit | hit | - |  |  |  |  |
| SAOUHSC_00554 | HxlB | hxlB | - |  |  |  |  |
| SAOUHSC_01451 | IlvA1 | ilvA1 | - |  |  |  |  |
| SAOUHSC_00153 | IpdC | ipdC | - |  |  |  |  |
| **SAOUHSC_03006** | **Lip** | **lip** |  | **-** |  | **+** |  |
| SAOUHSC_00844 | MetQ | metQ | + |  |  |  |  |
| SAOUHSC_00887 | MnhC | mnhC | + |  |  |  |  |
| SAOUHSC_01726 |  |  | + |  |  |  |  |
| **SAOUHSC_02927** | **Mqo2** | **mqo2** |  |  |  | **-** |  |
| **SAOUHSC_01759** | **MreC** | **mreC** |  |  |  | **+** |  |
| SAOUHSC_01361 | MsrR | msrR |  |  |  |  |  |
| **SAOUHSC_02919** | **PanB** | **panB** | **-** |  |  | **+** |  |
| **SAOUHSC_01145** | **PbpA** | **pbpA** |  |  |  | **+** |  |
| **SAOUHSC_01652** | **Pbp3** | **pbp3** | **+** | **-** |  |  |  |
| SAOUHSC_02377 | Pdp | pdp | - |  |  |  |  |
| **SAOUHSC_00051** | **Plc** | **plc** | **-** |  | **-** | **-** |  |
| **SAOUHSC_01972** | **PrsA** | **prsA** | **+** |  | **-** | **+** |  |
| **SAOUHSC_01008** | **PurE** | **purE** |  |  |  | **+** |  |
| **SAOUHSC_00257** | **EsxA** | **esxA** | **-** | **-** |  |  |  |
| SAOUHSC_00269 |  |  |  |  |  |  |  |
| SAOUHSC_00305 |  |  | - |  |  |  |  |
| SAOUHSC_00426 |  |  | + |  |  |  |  |
| SAOUHSC_00535 | CapD | capD |  |  |  |  |  |
| SAOUHSC_00997 |  |  |  |  |  |  |  |
| SAOUHSC_01193 | VfrB | vfrB |  |  |  |  |  |
| **SAOUHSC_01857** | **FtsK** | **ftsK** |  |  |  | **+** |  |
| SAOUHSC_02033 |  |  | - |  |  |  |  |
| SAOUHSC_02062 |  |  |  |  |  |  |  |
| SAOUHSC_02121 | CamS | camS |  |  |  |  |  |
| SAOUHSC_02308 | YsbT | ydbT |  |  |  |  |  |
| SAOUHSC_02791 | MuT | mutT | - |  |  |  |  |
| **SAOUHSC_00025** | **SasH** | **sasH** |  |  | **-** |  |  |
| SAOUHSC_01253 | SpoIIIE | spoIIIE |  |  |  |  |  |
| SAOUHSC_01901 | Tal | tal | - |  |  |  |  |
| **SAOUHSC_01337** | **Tkt** | **tkt** |  |  |  | **+** |  |
| SAOUHSC_01367 | TrpG | trpG |  |  |  |  |  |
| SAOUHSC_01701 | YqeG | yqeG |  |  |  |  |  |
| SAOUHSC_00878 | NdH | ndH | + |  |  |  |  |

^1^Rows in bold mark proteins/ genes, of which the level or expression, respectively, was influenced by inactivation of *sarA.*

^2^A plus means a positive influence of SarA on the transcription level of the respective gene or protein. A minus represents a negative influence of SarA on the transcription level of the respective gene or protein

Proteins are classified according to the SEED annotation [4].

**References**

[1] Dunman PM, Murphy E, Haney S, Palacois D, Tucker-Kellogg G et al. (2001) Transciption profiling-based identification of *Staphylococcus aureus* genes regulated by the *agr* and/or *sarA* loci. J Bacteriol 183:7341-7353.

[2] Jones RC, Deck J, Edmondson RD, Hart ME (2008) Relative quantitative comparisons of the extracellular protein profiles of *Staphylococcus aureus* UAMS-1 and its *sarA*, *agr*, and *sarA* *agr* regulatory mutants using one-dimensional polyacrylamide gel electrophoresis and nanocapillary liquid chromatography coupled with tandem mass spectrometry. J Bacteriol 190:5265-5278.

[3] Zielinska AK, Beenken KE, Mrak LN, Spencer HJ, Post GR et al. (2012) sarA-mediated repression of protease production plays a key role in the pathogenesis of *Staphylococcus aureus* USA300 isolates. Mol Microbiol 86:1183-1169.

[4] Overbeek R, Begley T, Butler RM, Choudhuri JV, Chuan HY et al. (2005) The subsystems approach to genome annotation and its use in the project to annotate 1000 genomes.Nucleic Acids Res 17:5691-5702
