# Supplementary material for: Sigma Factor SigB Is Crucial to Mediate Staphylococcus aureus Adaptation during Chronic Infections
Source: PLoS Pathog. 2015 Apr 29;11(4):e1004870. doi: 10.1371/journal.ppat.1004870 (PMC4414502; doi:10.1371/journal.ppat.1004870)
Supplement: S1 Fig — The growth curves of the wild-type strains LS1 (A) and SH1000 (B) and the corresponding mutants were performed in Müller-Hinton medium in order to determinate the Mean Generation Times (g) and growing rates (μ) (table; according the description in materials and methods). All the generation times of the different strains were not significantly different (ANOVA test, P>0.05). (C) The degree of hemolysis was evaluated in all the strains used in our study by measuring hemolytic activity spectrophotometrically to detect the release of hemoglobin from freshly isolated red blood cells (OD:570nm). S. aureus Wood 46 was used as a positive control. All the results are given relative to the values of S. aureus Wood 46 (hemolysis = 1). The graph represents the means of four independent experiments ±SD. All the groups were compared against the wild-type strains by ANOVA test, * P≤0.05. (PPTX) [file ppat.1004870.s004.pptx]

## Slide 1
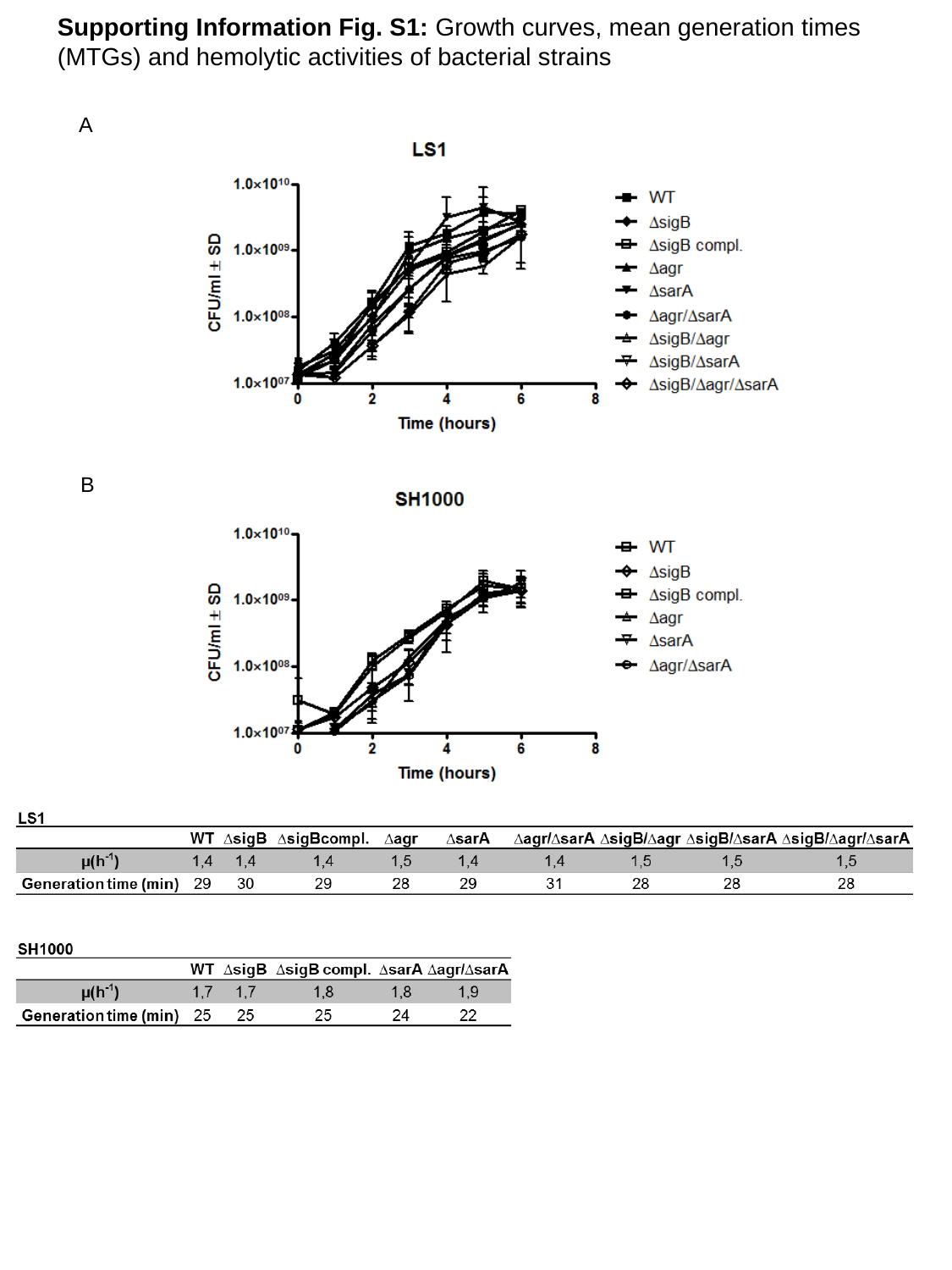

Supporting Information Fig. S1: Growth curves, mean generation times (MTGs) and hemolytic activities of bacterial strains
A
B

## Slide 2
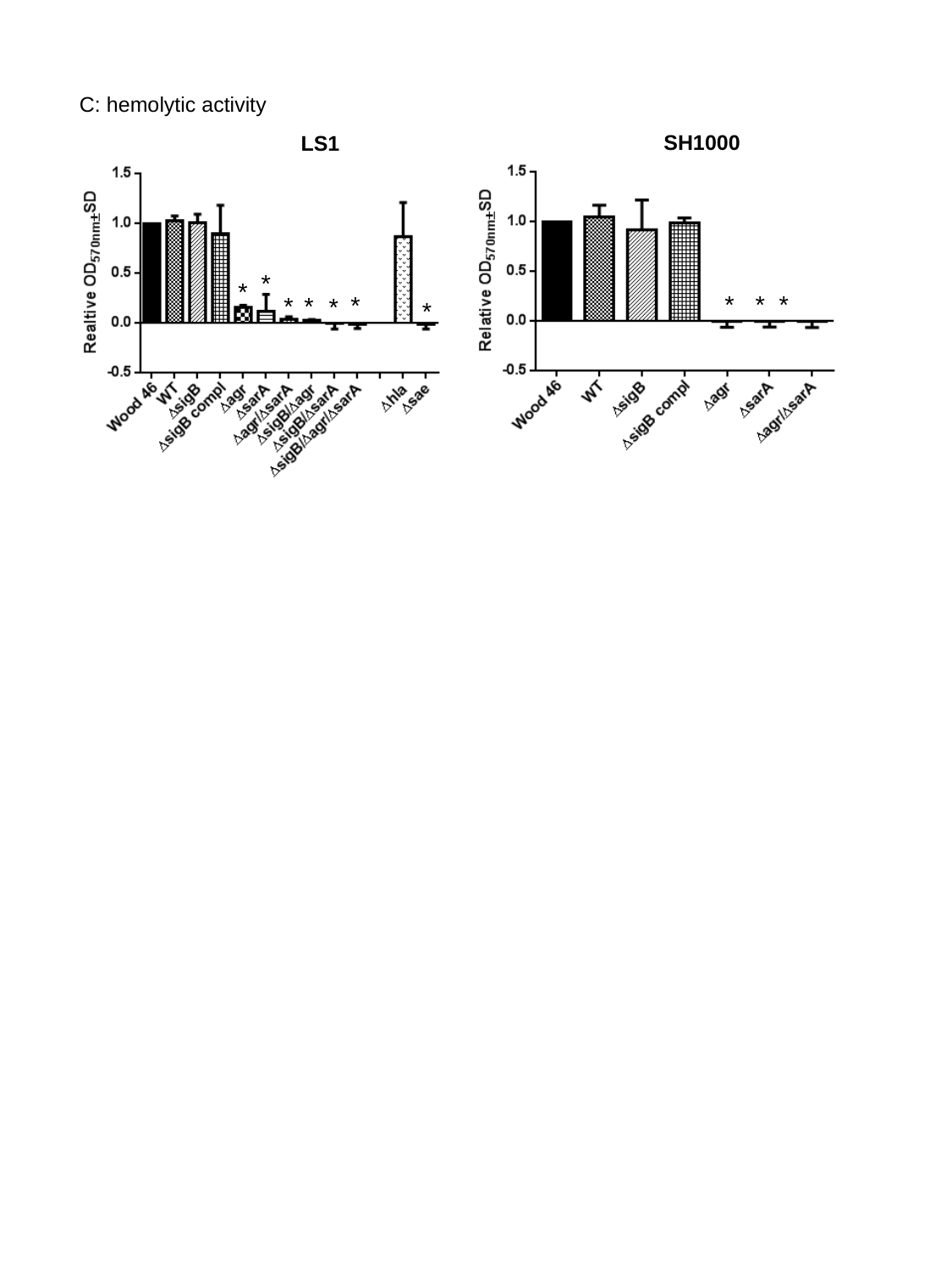

C: hemolytic activity
SH1000
LS1
*
*
* * *
*
*
*
*
*
