# Supplementary material for: Sigma Factor SigB Is Crucial to Mediate Staphylococcus aureus Adaptation during Chronic Infections
Source: PLoS Pathog. 2015 Apr 29;11(4):e1004870. doi: 10.1371/journal.ppat.1004870 (PMC4414502; doi:10.1371/journal.ppat.1004870)
Supplement: S3 Fig — The inflammatory effects of LS1, SH1000 and their mutants were evaluated on human osteoblasts. (A, B) Cultured osteoblasts were infected with LS1, SH1000 or their derivate mutants (MOI 50). After bacterial invasion (3 h) extracellular staphylococci were removed and infected cells were incubated with culture medium for 48 h. To analyze host cell response the changes in the expression of the chemokine CXCL-11 and CCL-5 were measured by real-time PCR. Results demonstrate the relative increase in gene expression, compared to unstimulated cells (control = 1). The values of all experiments represent the means ± SD of at least three independent experiments. (C, D) Measurement of chemokine release in cell culture supernatants by enzyme-linked immunosorbent assay (ELISA). Confluent human primary osteoblasts were infected with live LS1, SH1000 and their respective mutants (multiplicity of infection, 50) and incubated for 24 h as described in Materials and Methods. The conditioned media were analyzed for RANTES (regulated on activation of normal T cell expressed and secreted). Results are means ± SD of 2 independent experiments performed in duplicates. * P≤0.05 ANOVA test was used to compare the effects induced by the wild-type strains and the corresponding mutants. (PPTX) [file ppat.1004870.s006.pptx]

## Slide 1
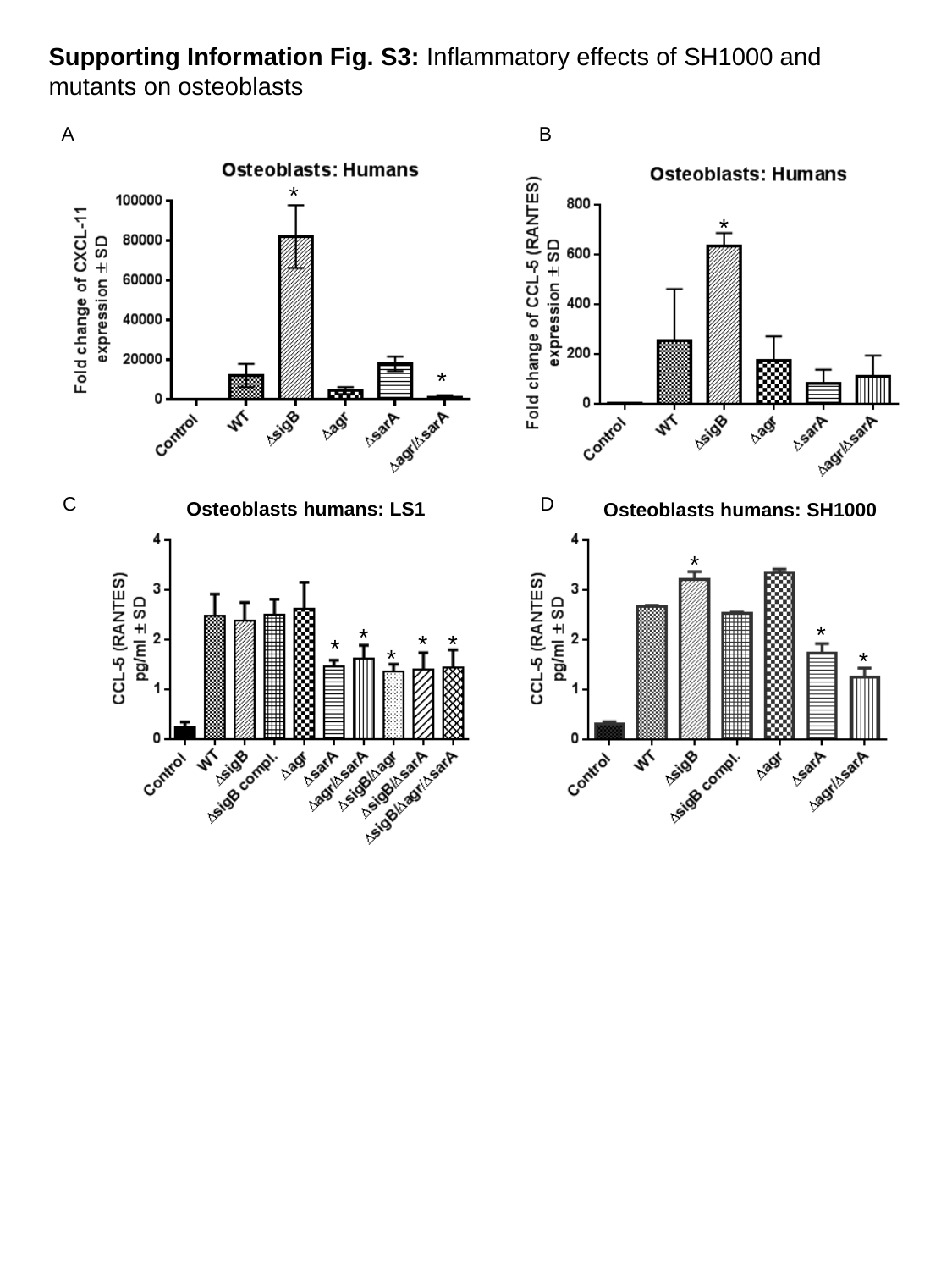

Supporting Information Fig. S3: Inflammatory effects of SH1000 and mutants on osteoblasts
A
B
*
*
*
C
D
Osteoblasts humans: LS1
Osteoblasts humans: SH1000
*
*
*
*
*
*
*
*
