# Supplementary material for: Sigma Factor SigB Is Crucial to Mediate Staphylococcus aureus Adaptation during Chronic Infections
Source: PLoS Pathog. 2015 Apr 29;11(4):e1004870. doi: 10.1371/journal.ppat.1004870 (PMC4414502; doi:10.1371/journal.ppat.1004870)
Supplement: S4 Fig — (A) The uptake of S. aureus wild-type strains LS1 and SH1000 were measured in human endothelial cells (HUVECs) and osteoblasts by plating cell lysates directly after infection and counting the CFU on the following day. (B) The cell activation of S. aureus wild-type strains LS1 and SH1000 were measure in osteoblasts and endothelial cells (HUVECs) by real time after 48h post infection. The values represent the means ± SD of three independent experiments performed in triplicate. * P≤0.05 t-test comparing the two cell types. (C, D) Cultured HUVECs were infected with S. aureus SH1000 or their derivate mutants (MOI 50). After bacterial invasion (3 h) extracellular staphylococci were removed by washing and lysostaphin treatment and infected cells were incubated with culture medium for 48 h. To analyze host cell response the changes in the expression of the chemokine CXCL-11 were measured by real-time PCR. Results demonstrate the relative increase in gene expression, compared to unstimulated cells (control = 1). The values of all experiments represent the means ± SD of at least three independent experiments. * P≤0.05 ANOVA test was used to compare the effects induced by the wild-type strains and the corresponding mutants. (PPTX) [file ppat.1004870.s007.pptx]

## Slide 1
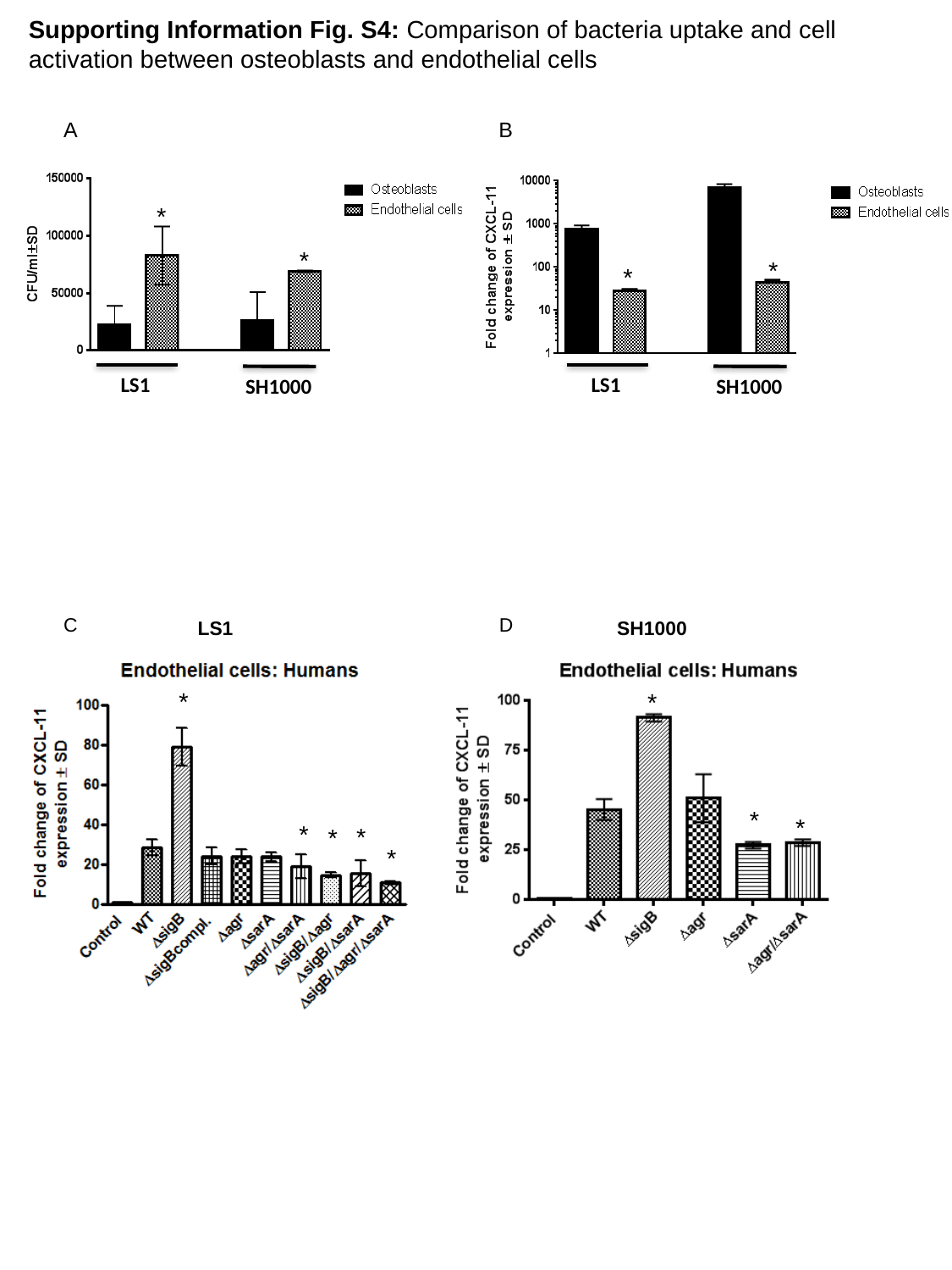

Supporting Information Fig. S4: Comparison of bacteria uptake and cell activation between osteoblasts and endothelial cells
B
A
*
*
*
*
LS1
LS1
SH1000
SH1000
C
D
LS1
SH1000
*
*
*
*
*
*
*
*
