# Supplementary material for: Sigma Factor SigB Is Crucial to Mediate Staphylococcus aureus Adaptation during Chronic Infections
Source: PLoS Pathog. 2015 Apr 29;11(4):e1004870. doi: 10.1371/journal.ppat.1004870 (PMC4414502; doi:10.1371/journal.ppat.1004870)
Supplement: S5 Fig — The initial intracellular counts of S. aureus wild-type strains LS1 and SH1000 and their respective mutants were measured in human osteoblasts by plating cell lysates directly after infection (time zero). The values represent the means ± SD of three independent experiments performed in triplicate. No significant differences were found between the different strains (ANOVA p>0,05). (PPTX) [file ppat.1004870.s008.pptx]

## Slide 1
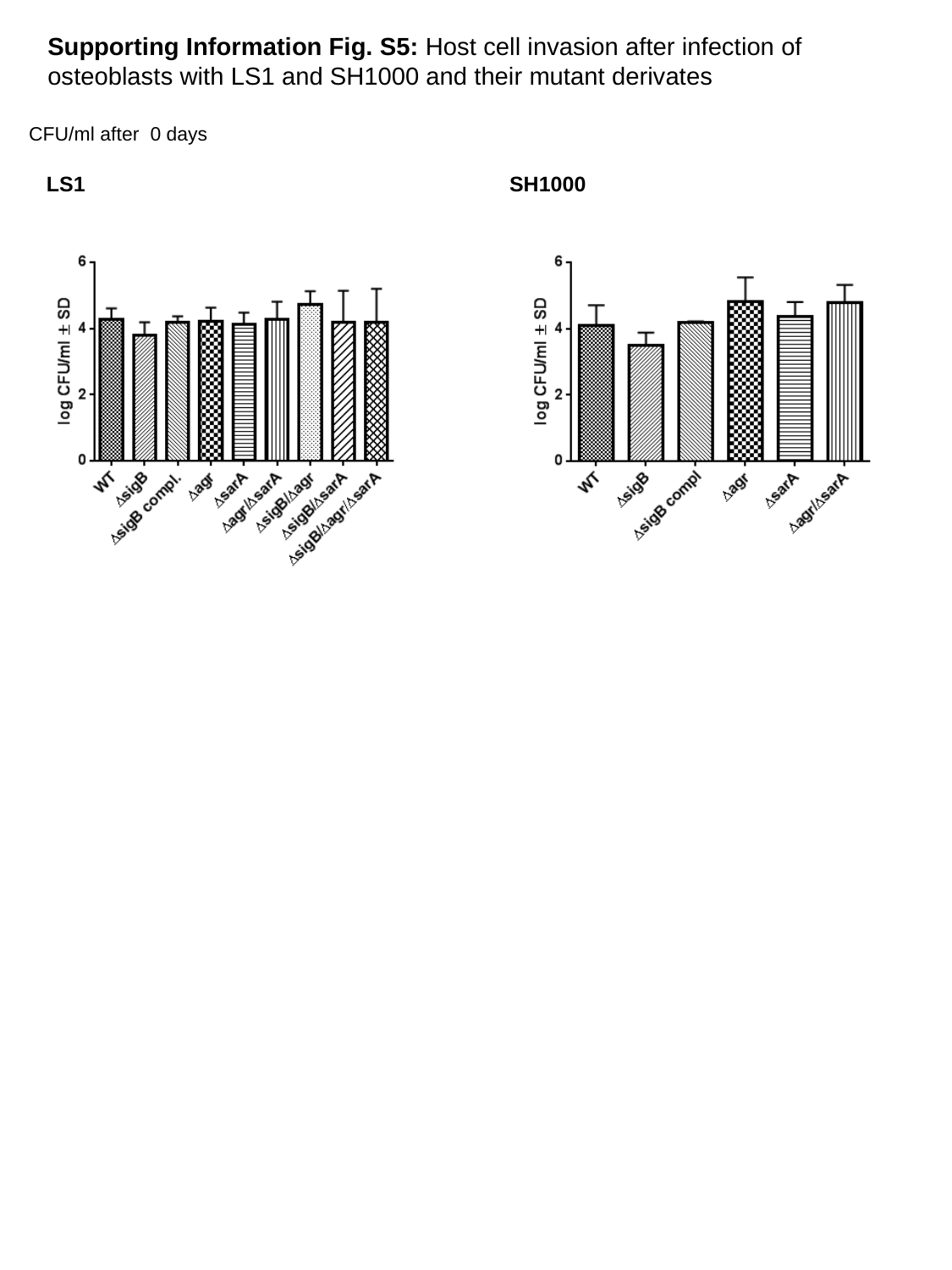

Supporting Information Fig. S5: Host cell invasion after infection of osteoblasts with LS1 and SH1000 and their mutant derivates
CFU/ml after 0 days
LS1
SH1000
