# Supplementary material for: Sigma Factor SigB Is Crucial to Mediate Staphylococcus aureus Adaptation during Chronic Infections
Source: PLoS Pathog. 2015 Apr 29;11(4):e1004870. doi: 10.1371/journal.ppat.1004870 (PMC4414502; doi:10.1371/journal.ppat.1004870)
Supplement: S6 Fig — (A) Osteoblasts were infected with LS1 (WT) and mutants as described before. After the lysostaphin step the infected cells were incubated with medium and cell death assays were performed by measuring the proportion of hypodiploid nuclei as described. These measurements were performed every 2 days after infection. (B, C) The rates of cell death after 1 and 9 days post infection is shown separately. The values of all experiments represent the means ± SD of at least three independent experiments. * P≤0.05 ANOVA test was used to compare the effects induced by the wild-type strain and the corresponding mutants. (PPTX) [file ppat.1004870.s009.pptx]

## Slide 1
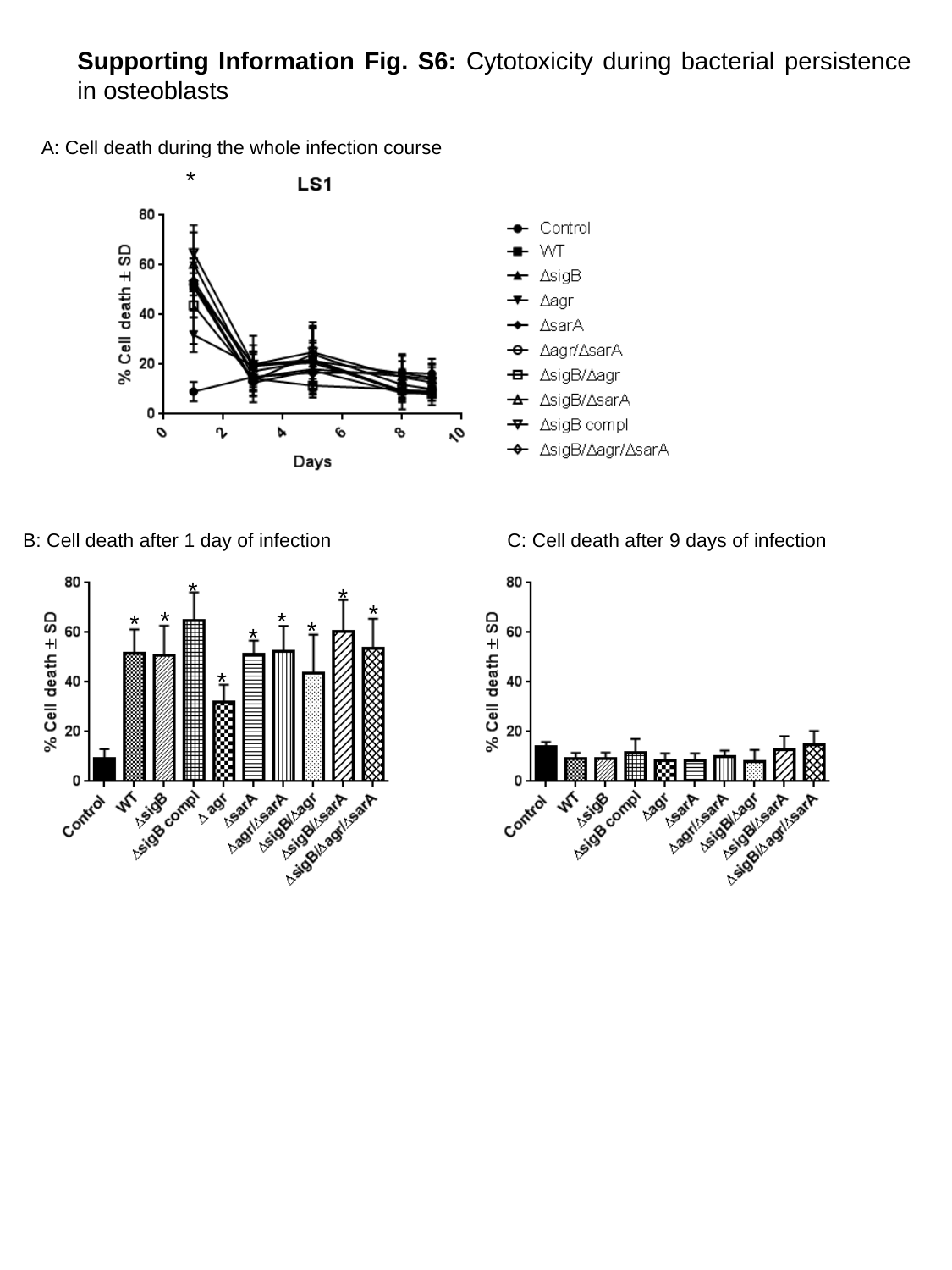

Supporting Information Fig. S6: Cytotoxicity during bacterial persistence in osteoblasts
A: Cell death during the whole infection course
*
B: Cell death after 1 day of infection
C: Cell death after 9 days of infection
*
*
*
*
*
*
*
*
*
