# Supplementary material for: Sigma Factor SigB Is Crucial to Mediate Staphylococcus aureus Adaptation during Chronic Infections
Source: PLoS Pathog. 2015 Apr 29;11(4):e1004870. doi: 10.1371/journal.ppat.1004870 (PMC4414502; doi:10.1371/journal.ppat.1004870)
Supplement: S7 Fig — (A) Cultured osteoblasts were infected with S. aureus strain SH1000 or the corresponding or complemented mutants as described and infected cells were analysed for up to 9 days. The numbers of viable intracellular persisting bacteria were determined every 2 days by lysing host cells, plating the lysates on agar plates and counting the colonies that have grown on the following day. (B) The results after 9 days are demonstrated separately. The results shown here are from osteoblast infection experiments, but similar results were obtained with endothelial cells. (C) The percentage of small and very small (SCV) phenotypes (<5 and <10-fold smaller than those of the wild-type phenotypes, respectively) recovered (between 200 and 500 colonies examined in each sample) were determined after 7 days p.i. The values of all experiments represent the means ± SD of at least three independent experiments. * P≤0.05 ANOVA test comparing the effects induced by the wild-type strain and the corresponding mutants. (D) Photographs of recovered colonies were performed after 7 days of infection of endothelial cells with strains LS1, LS1∆sigB or LS1∆sigBcompl. (PPTX) [file ppat.1004870.s010.pptx]

## Slide 1
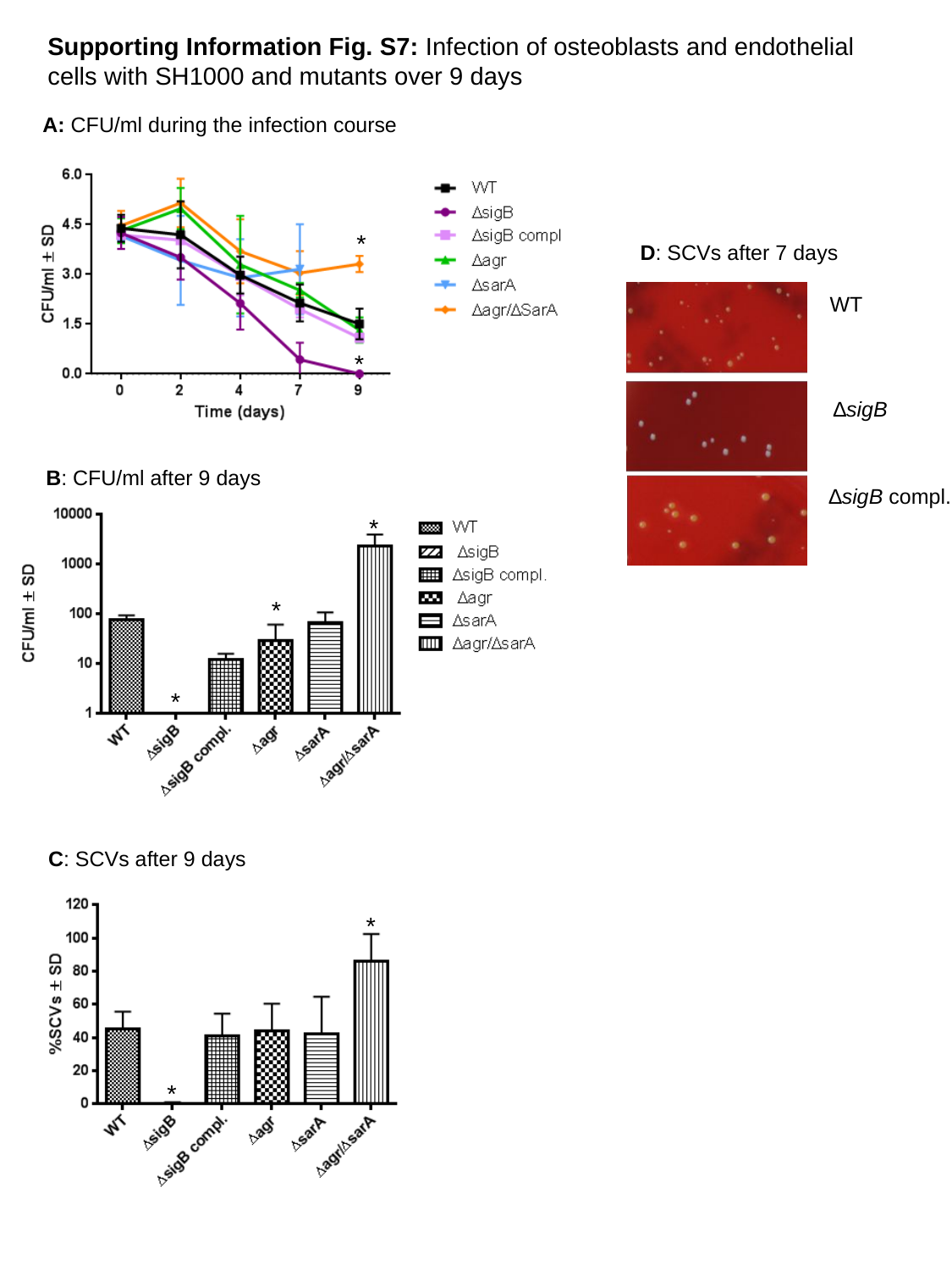

Supporting Information Fig. S7: Infection of osteoblasts and endothelial cells with SH1000 and mutants over 9 days
A: CFU/ml during the infection course
*
*
D: SCVs after 7 days
WT
∆sigB
B: CFU/ml after 9 days
∆sigB compl.
*
*
*
C: SCVs after 9 days
*
*
