# Supplementary material for: Sigma Factor SigB Is Crucial to Mediate Staphylococcus aureus Adaptation during Chronic Infections
Source: PLoS Pathog. 2015 Apr 29;11(4):e1004870. doi: 10.1371/journal.ppat.1004870 (PMC4414502; doi:10.1371/journal.ppat.1004870)
Supplement: S8 Fig — Cultured osteoblasts were infected with S. aureus wild-type strain LS1 or the corresponding mutants for sae (A, B) or hla (C, D) as described and infected cells were analysed for up to 9 days. (A, C) The numbers of viable intracellular persisting bacteria were determined every 2 days by lysing host cells, plating the lysates on agar plates and counting the colonies that have grown on the following day. The values represent the means ± SD of at least three independent experiments. *P≤0.05; T-test comparing the effects induced by the wild-type strain and the corresponding mutants did not reveal significant differences at any time point measured. (B, D) The cell death was monitored at each time point by FACS analysis. The values represent the means ± SD of at least three independent experiments. ANOVA was used to compare at different time points the effects of the wild-type strain and mutants on cell death induction in relation to control cells. * P≤0.05. (E) The cytokine release for LS1 (WT) and the corresponding mutants Δsae and Δhla was analyzed in primary osteoblast by ELISA as described in materials and methods. The differences between WT and mutants were not significant (ANOVA p>0,05). (PPTX) [file ppat.1004870.s011.pptx]

## Slide 1
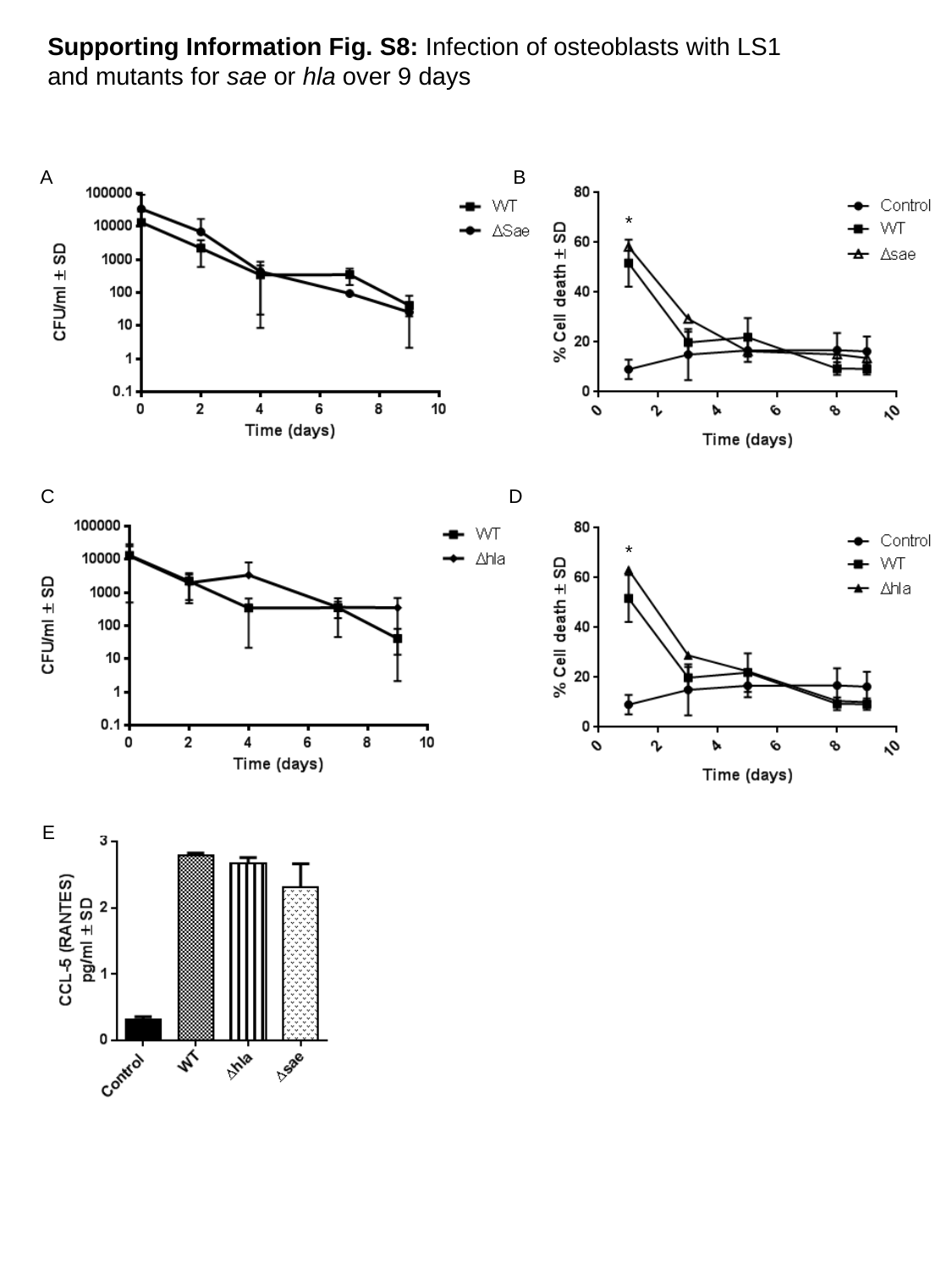

Supporting Information Fig. S8: Infection of osteoblasts with LS1 and mutants for sae or hla over 9 days
A
B
*
C
D
*
E
